# GM-CSF overexpression after influenza a virus infection prevents mortality and moderates M1-like airway monocyte/macrophage polarization

**DOI:** 10.1186/s12931-017-0708-5

**Published:** 2018-01-05

**Authors:** E. Scott Halstead, Todd M. Umstead, Michael L. Davies, Yuka Imamura Kawasawa, Patricia Silveyra, Judie Howyrlak, Linlin Yang, Weichao Guo, Sanmei Hu, Eranda Kurundu Hewage, Zissis C. Chroneos

**Affiliations:** 1 Department of Pediatrics, Pennsylvania State University College of Medicine, Hershey, PA USA; 2 Pulmonary Immunology and Physiology Laboratory, Pennsylvania State University College of Medicine, Hershey, PA USA; 3 Departments of Pharmacology & Biochemistry and Molecular Biology, Institute for Personalized Medicine, Pennsylvania State University College of Medicine, Hershey, PA USA; 4 Departments of Medicine and Public Health Sciences, Pennsylvania State University College of Medicine, Hershey, PA USA; 5 Department of Microbiology and Immunology, Pennsylvania State University College of Medicine, Hershey, PA USA

**Keywords:** Influenza, GM-CSF, Macrophage, Alveolar, Exudative, Pneumonia, RNA-seq, Interferon

## Abstract

**Background:**

Influenza A viruses cause life-threatening pneumonia and lung injury in the lower respiratory tract. Application of high GM-CSF levels prior to infection has been shown to reduce morbidity and mortality from pathogenic influenza infection in mice, but the mechanisms of protection and treatment efficacy have not been established.

**Methods:**

Mice were infected intranasally with influenza A virus (PR8 strain). Supra-physiologic levels of GM-CSF were induced in the airways using the double transgenic GM-CSF (DTGM) or littermate control mice starting on 3 days post-infection (dpi). Assessment of respiratory mechanical parameters was performed using the flexiVent rodent ventilator. RNA sequence analysis was performed on FACS-sorted airway macrophage subsets at 8 dpi.

**Results:**

Supra-physiologic levels of GM-CSF conferred a survival benefit, arrested the deterioration of lung mechanics, and reduced the abundance of protein exudates in bronchoalveolar (BAL) fluid to near baseline levels. Transcriptome analysis, and subsequent validation ELISA assays, revealed that excess GM-CSF re-directs macrophages from an “M1-like” to a more “M2-like” activation state as revealed by alterations in the ratios of CXCL9 and CCL17 in BAL fluid, respectively. Ingenuity pathway analysis predicted that GM-CSF surplus during IAV infection elicits expression of anti-inflammatory mediators and moderates M1 macrophage pro-inflammatory signaling by Type II interferon (IFN-γ).

**Conclusions:**

Our data indicate that application of high levels of GM-CSF in the lung after influenza A virus infection alters pathogenic “M1-like” macrophage inflammation. These results indicate a possible therapeutic strategy for respiratory virus-associated pneumonia and acute lung injury.

**Electronic supplementary material:**

The online version of this article (10.1186/s12931-017-0708-5) contains supplementary material, which is available to authorized users.

## Author summary

Using a transgenic mouse model where we can induce GM-CSF in the airways (BAL levels approximately 300 pg/mL) of WT mice with doxycycline administration during active influenza infection, we achieve levels of GM-CSF that prevent mortality from lethal influenza A virus infection. The GM-CSF excess rescued mice from an acute respiratory distress syndrome (ARDS)-like illness and stabilized lung mechanical parameters, improved clearance of exudate protein, and disconnected M1 activation by type II IFNγ.

## Background

Each year, influenza A virus (IAV) affects a significant proportion of the population [1] and causes pathologic changes both through direct cellular toxicity causing desquamation, de-ciliation, and cell death, and through indirect effects by stimulating an anti-viral immune response leading to collateral injury [2]. This combination can lead to an ARDS-like syndrome characterized by increased capillary leak, oxygen diffusion difficulty and ventilation/perfusion mismatch [1]. Immune strategies that protect the host’s lung function while still allowing for an adequate immune response to clear the viral load and resolve virus-induced pneumonia are needed. A number of pre-clinical studies have tested prophylactic GM-CSF both as vaccine adjuvant and local supplementation against IAV infection with encouraging results [3–6].

The effect of local elevation of GM-CSF on IAV infection in the lung has been investigated in transgenic models with expression of GM-CSF under the control of constitutive or doxycycline-inducible promoters in lungs of alveolar or small airway epithelial cells of GM-CSF knockout (*csf2*^*−/−*^) mice [3, 4]. Differential effects on morbidity and mortality from IAV infection in these studies was associated with increased alveolar macrophage (AM) numbers in the constitutive GM-CSF expression models [3, 5] and AM differentiation in the GM-CSF-inducible model [4]. Differential results on morbidity and survival were also obtained after prolonged or brief administration of supra-physiological levels of GM-CSF before or at the onset of IAV infection [6].

The question of whether therapeutic administration of GM-CSF to the airways after establishment of the infection would confer protection has never been addressed. In this study we use a more clinically relevant model to examine whether supra-physiologic levels of GM-CSF in the airways, induced after IAV infection at the peak of virus replication, provided therapeutic benefit. Using GM-CSF-inducible mice on the WT C57BL/6 genetic background we show that airway GM-CSF over-expression starting at 3 days post infection (dpi) provides protection from mortality and prevents the degeneration of multiple lung mechanical properties. To examine the mechanism of protection conferred by therapeutic GM-CSF levels, we measured respiratory and biochemical parameters of lower airway disease, and analyzed the transcriptome of FACS-sorted AMs and exudative macrophages (EM) from IAV-infected mice. Our findings demonstrate that GM-CSF restores proteostasis of exudate proteins and redirects responsiveness of AMs and EMs from an M1-like to an M2-like activation state, and prevents mortality from influenza-induced ARDS.

## Methods

### Animals and infections

The double transgenic GM-CSF (DTGM) mice were bred as previously described [4], but this time on the wild-type C57BL/6 J background. Littermate control (LM) mice were defined as being single transgenic littermates of DTGM mice that were only positive for the SCGB1A1-rTA and thereby did not have the CMV-GM-CSF gene, which may potentially be virally induced in the absence of tetracycline (doxycycline) [7]. DTGM mice and LM controls were exposed to 1 mg/mL doxycycline in drinking water, and doxycycline-containing drinking water was replenished every 2–3 days. Both male and female mice were used for all experiments; all mice were sex- and age-matched to control mice. The influenza strain A/Puerto Rico/8/34 (PR8) virus was a kind gift of Dr. Kevan Hartshorn, and was grown in the chorio-allantoic fluid of ten (10) day old specific pathogen free avian supplies (SPAFAS) chicken eggs purchased from Charles River Laboratories (Wilmington, MA) and purified on a discontinuous sucrose gradient as previously described [8]. Mice were anesthetized with ketamine/xylazine and intranasally (i.n.) infected with IAV virus in 40 μL of PBS. Mice were infected in a BSL2 biosafety cabinet and housed within filter-top micro-isolator cages in the Pulmonary Immunology and Physiology (PIP) core, a BSL2 facility in the Department of Comparative Medicine’s animal facility at Penn State University College of Medicine. Mice were observed at least twice daily during infections to assess morbidity and mortality. Based on our experience at our facility in the last 2 years and the variable clinical presentation of the influenza infection, we used other metrics to monitor morbidity in addition to mouse body weight curves [9]. Mice that exhibited immobility, ruffled hair, and labored breathing that had no chance of recovery, coinciding with approximately 30% of body weight loss, were euthanized by ketamine/xylazine overdose and cervical dislocation, and counted as dead. Alternatively, mice that were sleeping but had normal breathing and body appearance, i.e., no ruffled hair or labored breathing, reached up to 35-40% body weight loss and then began to recover normally. Mice with favorable prognosis but with 30% body weight loss or greater were provided supportive care with food and hydrated gel packs at the bottom of the cage. We have not found a pattern of clinical disease specific to mouse genotype or gender in untreated mice.

### Mouse ventilation and the measurement of oxygen saturation and lung mechanics

Mice were anesthetized with ketamine/xylazine (130 mg/kg and 10 mg/kg, i.p., respectively). The trachea was cannulated via tracheostomy with a 19G blunt needle and the cannula was secured in place with a suture. Mice were kept sedated using isoflurane inhalation (maintenance dosing, 1-5% of inspired air) through the flexiVent and were paralyzed with 1 mg/kg vecuronium i.p. Mice were ventilated using baseline settings of positive end expiratory pressure (PEEP) 3 cm H_2_O, tidal volumes (Vt) 10 mL/kg, respiratory rate (RR) 150 breaths per minute (bpm) and an fraction of inspired oxygen (FiO2) of 0.21. Oxygen saturations were measured using the MouseOx Plus pulse oximeter (Starr Life Sciences, Oakmont, PA, USA) via the thigh sensor. Lung mechanic parameters were generated from the flexiVent rodent ventilator using the forced oscillation technique as previously described [10].

### Bronchoalveolar lavage (BAL) protein measurements

BAL samples were collected as previously described, and after centrifugation at 150 g for 10 min, BAL supernatants were removed and immediately frozen at −80°C until batch analysis. Proteins were measured with kits as detailed in Additional file 1: Table S1. ELISA plate absorbance was measured at 450 nm with a SpectraMax M2 UV/Vis/Fluorescence 96-384 plate reader (Molecular Devices, Sunnyvale, CA). Cytokines were measured by ELISA as described above or by ProcartaPlex cytokine & chemokine 36-plex mouse panel 1A (Thermo Fisher Scientific) via Luminex Magpix multiplex array (Luminex).

### Quantitative RT-PCR for IAV M1 copies per lung

After IAV infection, the entire lung was removed from each mouse and placed in 2 mL of TRIzol (Thermo Fisher Scientific, Waltham MA), weighed, homogenized on ice using a polytron homogenizer for 15-30s intervals, and frozen in aliquots at -80 °C until RNA extraction. DNA was then extracted using chloroform and RNA was precipitated using isopropanol. Quantitative RT-PCR for IAV M1 copies per lung was performed as previously described [11] using the following primers: influenza A/8/Puerto Rico/34 M1 gene sense: 5’-AAGACCAATCCTGTCACCTCTGA-3′ and antisense: 5’ CAAAGCGTCT-ACGCTGCAGTC -3′ primers, and the Mediator Probe sequence: 5′- /56 FAM/TTTGTGTTCACGCTC-ACCGT/36-TAMSp/ -3′. Data are expressed as M1 viral copies per lung.

### Flow cytometric cell surface staining and sorting

Single cell suspensions were prepared from BAL and lung as described in Supplemental Methods. Single cell suspensions from lung digests were placed at 4°C and then surface stained in Hank’s Buffered Saline Solution (HBSS) with 3% FBS with fluorochrome-conjugated monoclonal antibodies (Additional file 2: Table S2), and then stained with a fixable viability dye. For FACS-sorting, BAL cells were recovered and placed at 4°C and then surface stained in Hank’s Buffered Saline Solution (HBSS) with 3% FBS with fluorochrome-conjugated monoclonal antibodies (Additional file 2: Table S2) and 7-aminoactinomycin D (7-AAD) was used to assess viability just prior to acquisition. All flow cytometric data were collected in the Penn State Hershey Flow Cytometry Core Facility using an LSR II (Becton Dickinson, BD) instrument, and all FACS-sorting was performed using a FACSAria (BD) High Speed cell sorter. Cells were sorted directly into RNA-Bee to isolate RNA for further RNA-sequencing (RNA-seq). All FACS data analysis was performed using FlowJo version 9.9 (Treestar, Mountain View, CA).

### RNA preparation, library construction and sequencing

RNA was phase separated using chloroform and the aqueous phase containing RNA was removed following centrifugation and precipitated overnight at −20°C using ice cold isopropanol. RNA was washed with 75% ethanol then solubilized in RNase-free water. Optical density values of extracted RNA were measured using NanoDrop (Thermo Fisher Scientific) to confirm an A260:A280 ratio above 1.9. RNA integrity number (RIN) was measured using BioAnalyzer (Agilent) RNA 6000 Pico Kit to confirm RIN above 7. The cDNA libraries were prepared using the SMARTer® Ultra® Low Input RNA Kit for Sequencing - v3 (Clontech) followed by Nextera XT DNA Library Prep Kit (Illumina) as per the manufacturer’s instructions. The unique barcode sequences were incorporated in the adaptors for multiplexed high-throughput sequencing. The final product was assessed for its size distribution and concentration using BioAnalyzer High Sensitivity DNA Kit (Agilent Technologies) and Kapa Library Quantification Kit (Kapa Biosystems). The libraries were diluted to 2 nM in EB buffer (Qiagen) and then denatured using the Illumina protocol. The denatured libraries were diluted to 10 pM by pre-chilled hybridization buffer and loaded onto TruSeq SR v3 flow cells on an Illumina HiSeq 2500 (Illumina) and run for 50 cycles using a single-read recipe (TruSeq SBS Kit v3, Illumina) according to the manufacturer’s instructions. Illumina CASAVA pipeline (released version 1.8, Illumina) was used to obtain de-multiplexed sequencing reads (fastq files) passed the default purify filter. Additional quality filtering used FASTX-Toolkit (http://hannonlab.cshl.edu/fastx_toolkit) to keep only reads that have at least 80% of bases with a quality score of 20 or more (conducted by fastq_quality_filter function) and reads left with 10 bases or longer after being end-trimmed with reads with a base quality score of b20 (conducted by fastq_quality_trimmer function).

### RNA-sequencing alignment and differential gene expression analysis

A bowtie2 index was built for the mouse reference genome (GRCm38) using bowtie version 2.1.0. The RNA-seq reads of each of the 38 samples were mapped using TopHat version 2.0.9 [12] supplied by Ensembl annotation file; GRCm38.78.gtf. Gene expression values were computed using fragments per kilobase per million mapped reads (FPKM). Differential gene expression was determined using Cuffdiff tool which is available in Cufflinks version 2.2.1 [13] supplied by GRCm38.78.gtf. Normalization was performed via the median of the geometric means of fragment counts across all libraries, as described in Anders and Huber [14]. Statistical significance was assessed using a false discovery rate threshold of 0.05.

### Functional annotation enrichment analysis

We arbitrarily chose to further analyze the 5% most highly expressed gene transcripts in AM or EM cell populations from IAV-infected DTGM or LM mice using Ingenuity Pathway Analysis (IPA, www.qiagen.com/ingenuity) to identify upstream signaling pathways. Significance was measured by Fisher’s exact test with a q < 0.2 cut-off.

### Statistics

All statistical analysis was performed using JMP 12.0.1 software (SAS, Cary, NC). Normally distributed data were analyzed using student’s t-test, and non-normally distributed data using Wilcoxon signed-rank test. Survival analysis was calculated by using the log-rank test. All data points are means ± standard error of the mean (SEM) unless otherwise stated. Graphs were created using Prism 6 for Mac OS X (GraphPad, La Jolla, CA).

## Results

### Doxycycline-inducible airway GM-CSF over-expression confers protection against severe IAV infection

To characterize the pathogenicity of our H1N1 PR8 IAV preparation virus, wild-type C57BL/6 J mice (The Jackson Laboratory, MA) were purchased and we determined the lethal dose 50% (LD_50_) of our PR8 IAV preparation. Female wild-type mice were much more susceptible than males with an LD_50_ approximately 5-fold lower than males (728 vs. 3728 FFU, female and males respectively, Additional file 3: Figure S1A-D).

**Fig. 1:**
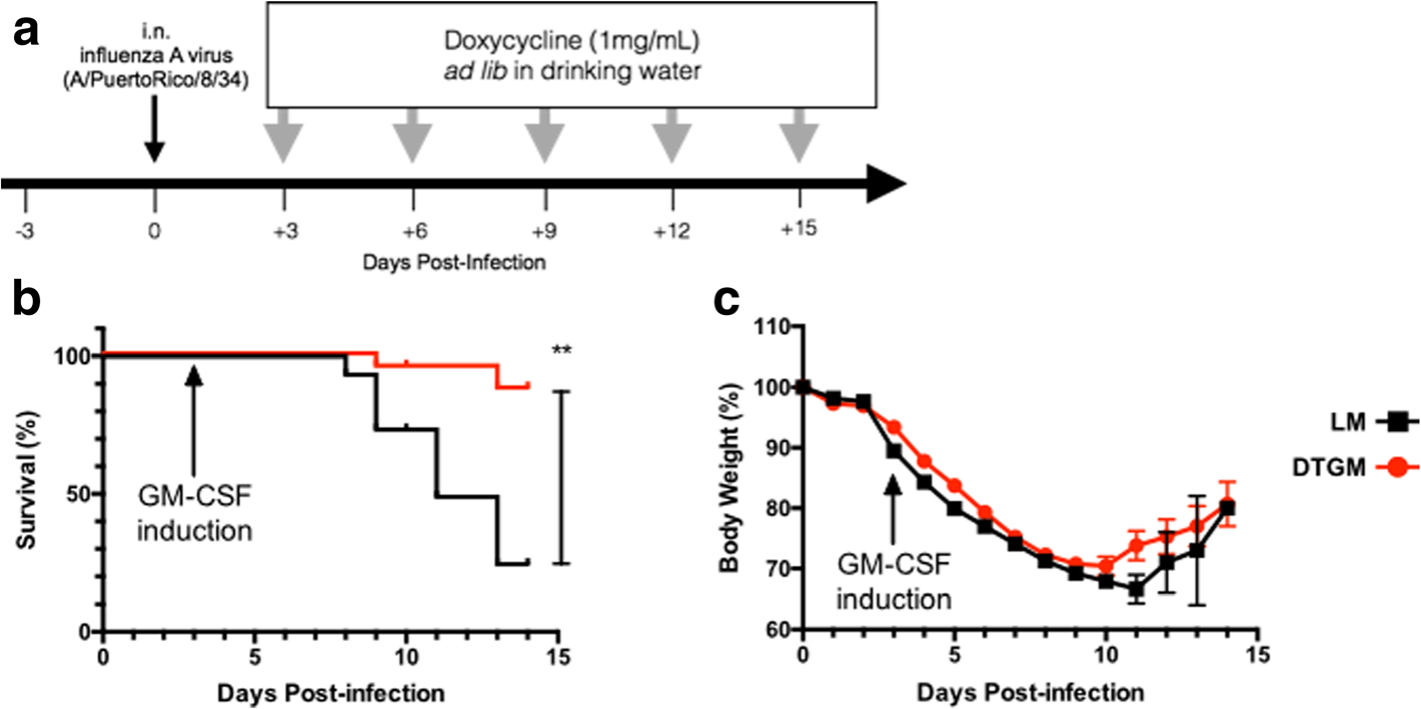
Therapeutic model of GM-CSF during IAV infection using an inducible airway GM-CSF over-expression transgenic mouse model, and effects on survival and body mass during IAV infection. To simulate a therapeutic model of GM-CSF administration doxycycline was administered to both DTGM and LM control mice starting 3 days after i.n. infection with PR8 IAV. Doxycycline-containing water was protected from light and changed every three days (**a**). DTGM (*n* = 23, red circles/lines) and LM control (*n* = 15, black squares/lines) mice were administered approximately 2 LD_50_ of IAV PR8 virus i.n. and administered doxycycline in water starting on +3 dpi, and the effects on survival and body weight are shown. Mice were euthanized if they lost >30% body weight and were moribund. GM-CSF over-expression (DTGM mice) conferred a significant survival benefit (**b**) but not a significant effect on weight loss/recovery (**c**) as compared to wild-type levels (LM mice). Results shown represent three independent experiments (**p < 0.005)

Airway GM-CSF levels were conditionally increased following IAV infection using a doxycycline inducible promoter in the DTGM mouse model, formerly named the *tet-GM*^*+/+*^*,* as previously described [4]. In this conditional transgenic mouse model GM-CSF is expressed and secreted by airway club cells via the club cell 10 (CC10, *Scgb1a1*) promoter after oral administration of doxycycline (1 mg/mL in water ad libitum) (Fig. 1a). Importantly, in the absence of infection, BAL fluid levels of GM-CSF in DTGM mice are near the limit of detection, similar to littermate controls (Additional file 4: Figure S2a), and their alveolar macrophages appear identical by multi-parameter flow cytometry. Once doxycycline is administered, BAL levels of GM-CSF peak after approximately 48 h reaching levels of approximately 500 pg/mL in 2.5 mL of recovered BAL fluid and in preliminary experiments the DTGM mice were either administered or not administered doxycycline to create a condition of elevated vs. wild-type levels of airway GM-CSF, respectively [4]. However, these preliminary experiments demonstrated that low levels of GM-CSF from the *Scgb1a1* promoter in DTGM mice were endogenously induced by interferons during IAV infection (Additional file 4: Figure S2A), a finding that has previously been reported [15]. Therefore, all subsequent experiments compared the DTGM to LM groups, both exposed to doxycycline, to examine the effect of elevated (DTGM) as opposed to wild-type (LM) levels of airway GM-CSF, while also controlling for any off-targets effects of doxycycline.

To address our research question of whether “treatment” with GM-CSF during severe IAV infection would improve survival, DTGM and LM mice were infected i.n. with approximately 1 LD_50_ (differential dosing based on sex) of PR8 IAV and were administered doxycycline in drinking water. GM-CSF overexpression (DTGM) conferred a significant survival advantage as compared to wild-type levels (LM, Fig. 1b, **p < 0.005). Weight loss and recovery were similar in the two groups, however, because of survivor bias likely artificially elevating the average weights of surviving LM mice (Fig. 1c). Of note, doxycycline treatment of LM mice had no effect on survival whereas doxycycline-untreated DTGM mice demonstrated a survival advantage over untreated LM mice, suggesting that even low levels of GM-CSF can confer some survival benefit (Additional file 4: Figure S2B).

### GM-CSF does not improve oxygenation but prevents deterioration of several lung mechanical parameters

Lower respiratory tract IAV infection can lead to impaired oxygenation due to V:Q mismatch and decrease lung compliance due to the infiltration of inflammatory cells and an increase in lung water weight [16]. Given the ability of GM-CSF to confer survival, we expected elevated GM-CSF levels to improve arterial oxygen saturations (% SpO_2_). However, GM-CSF did not significantly increase median oxygen saturations (% SpO_2_) levels as compared to LM mice at either 7 or 10 dpi (data not shown).

**Fig. 2:**
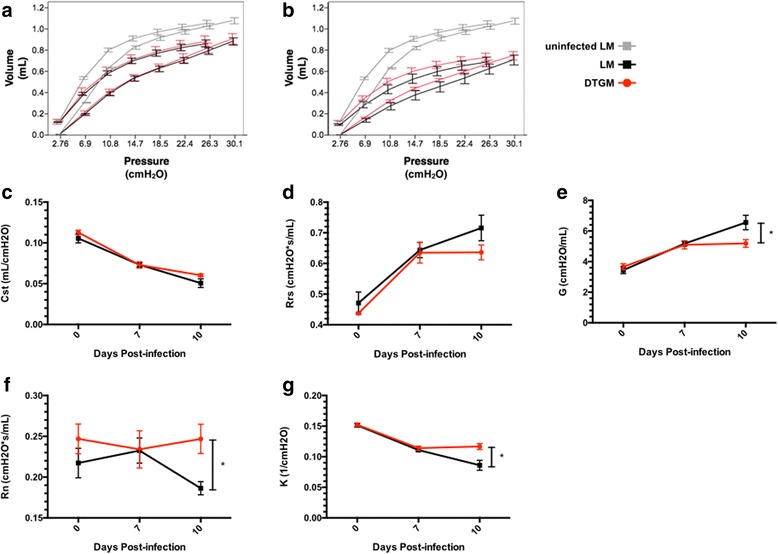
Effects of supra-physiologic levels of GM-CSF on lung mechanical properties during IAV infection. Pressure-volume (PV) curves showing the mean±SEM of lung volume (mL) of each group, LM (black) and DTGM (red) at each indicated preprogramed pressure (cmH_2_O) at 7 dpi (**a**) and at 10 dpi (**b**)(*n* = 5-9 mice per group per time point). The PV curves of uninfected mice (gray) are shown on each graph for comparison. Shown are the lung mechanical parameters of static compliance (Cst, mL*cmH_2_O^−1^)(**c**), total respiratory system resistance (Rrs, cmH_2_O*s^−1^*mL^−1^)(**d**), tissue damping or peripheral airway resistance (G, cmH_2_O*s^−1^*mL^−1^)(**e**),Newtonian or central airway resistance (Rn, cmH_2_O*s^−1^*mL^−1^)(**f**), and the curvature of the deflation limb of the PV curve (K, cmH_2_O^−1^)(**g**). Results shown represent three independent experiments, *n* = 4-10 mice per group per time point (*p < 0.05)

To gain insight into whether GM-CSF conferred any lung mechanical benefits, lung mechanics scans were performed by the forced oscillation technique and PV loop curves were generated (Fig. 2a, b). As expected, the PV curve flattens with IAV infection due to decreased static compliance (Cst), but we were surprised that compliance continued to fall from days 7 to 10 (Fig. 2a-c). While GM-CSF did not affect Cst (Fig. 2c) or total system resistance (Rrs, Fig. 2d), DTGM mice demonstrated less tissue damping or peripheral airway resistance (G, cmH_2_O/mL, Fig. 2e), and significant preservation of Newtonian or central airway resistance (Rn, cmH_2_O*s/mL, Fig. 2f) and curvature of the deflation limb of the PV curve, a measure of maintenance of alveoli and small airway recruitment (K, 1/cmH_2_O, Fig. 2g) at 10 dpi.

### GM-CSF decreases BAL protein levels late during infection but does not alter viral load

**Fig. 3:**
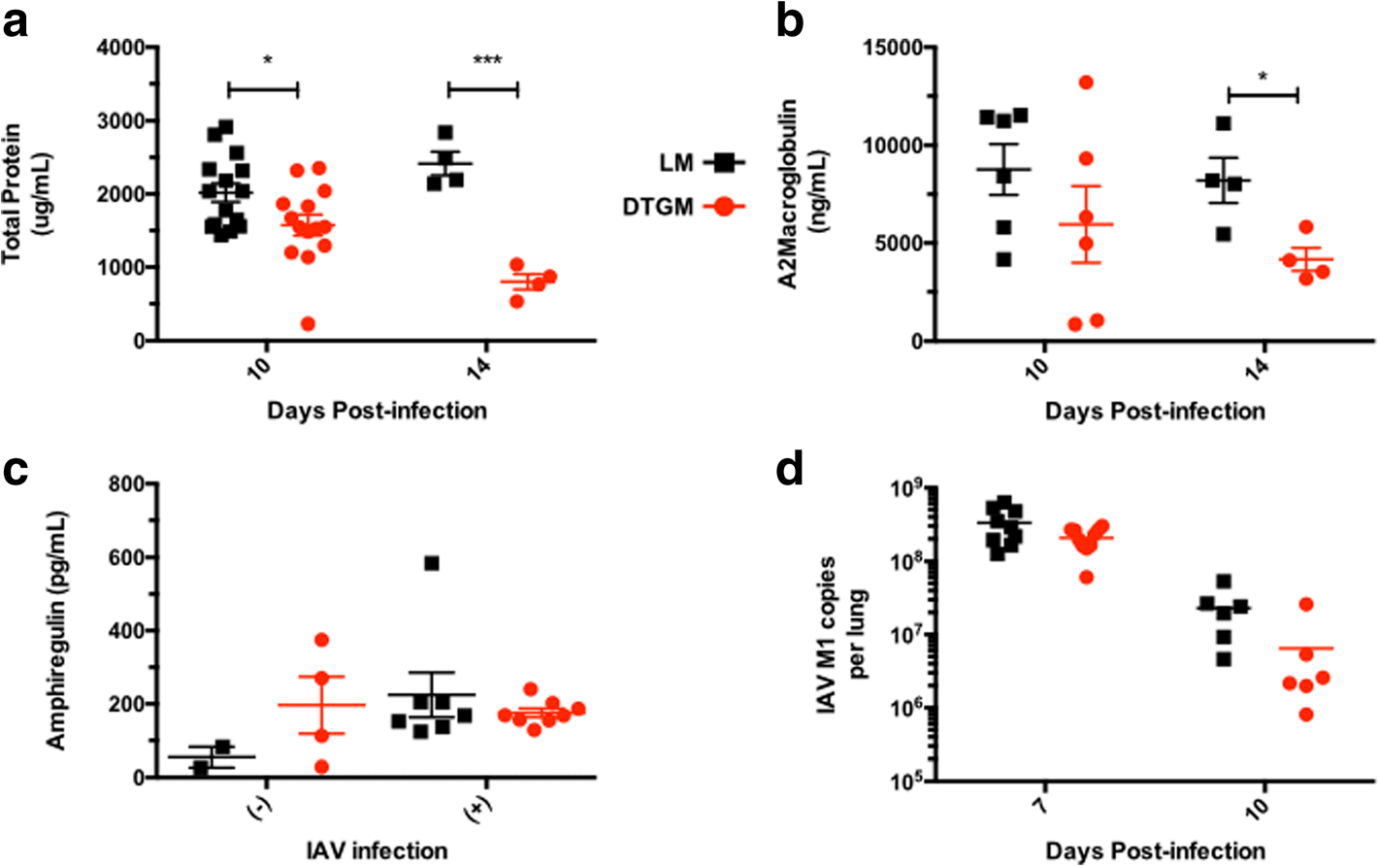
Effects of supra-physiologic levels of GM-CSF on bronchoalveolar lavage fluid content and influenza A virus load. BAL fluid was recovered from mice at indicated time points after IAV infection and total protein (**a**) was measured by BCA assay, while BAL concentrations of alpha-2-macroglobulin (**b**) and amphiregulin (**c**) levels were quantitated by ELISA. The number of influenza A virus matrix protein (M1) transcripts was quantitated from whole lung by RT-PCR (**d**). Results shown represent three independent experiments (*p < 0.05, ***p < 0.0005)

Given that two of the lung mechanical parameters that are maintained by GM-CSF, K and G, are correlated with dynamic processes at the small airway or alveolar level, namely alveolar size [17] and changes in tissue physical properties of small airways [18] respectively, and which can change with lung interstitial edema [19], we hypothesized that GM-CSF may improve lung capillary barrier function and/or enhance alveolar fluid clearance. As surrogate of alveolar fluid content, we measured the concentration of total protein in BAL fluid and found that GM-CSF overexpression decreased BAL fluid total protein levels at 10 (peak inflammation) and 14 dpi (early resolution phase) (Fig. 3a). To further investigate this difference in BAL fluid protein content, we examined the concentration of various serum and lung-specific proteins including mouse serum albumin (69 kDa), as well as two larger proteins, alpha-2-macroglobulin (180 kDa monomer, 720 kDa tetramer) and immunoglobulin M (IgM, 194 kDa monomer, 970 kDa pentamer) as markers of capillary leak [20]. GM-CSF significantly decreased alpha-2-macroglobulin levels at 14 dpi (Fig. 3b), but did not significantly decrease other markers of capillary leak including albumin or IgM (Additional file 5: Figure S3A-B). We also directly assayed the lung epithelial barrier function with FITC-labeled dextran (MW 10,000), but surprisingly no differences in epithelial barrier function could be detected at 10 dpi (data not shown).

Additionally, we also investigated whether GM-CSF overexpression during IAV increased BAL levels of the epidermal growth factor family member, amphiregulin. GM-CSF overexpression in uninfected mice elevated levels of amphiregulin, though IAV infection also induced amphiregulin and GM-CSF did not further increase these levels (Fig. 3c). Lastly, we assessed whether elevated GM-CSF levels during active infection affected viral clearance. At 7 dpi, the peak of virus levels in our model, we recovered 2-3 × 10^8^ M1-copies total lung copies of IAV PR8 matrix 1 (M1) via RT-PCR and the viral copies decreased to 0.8-2 × 10^7^ by 10 dpi, though there was no statistically significant difference with GM-CSF overexpression (Fig. 3d).

### Effect of GM-CSF overexpression on airway macrophages

**Fig. 4:**
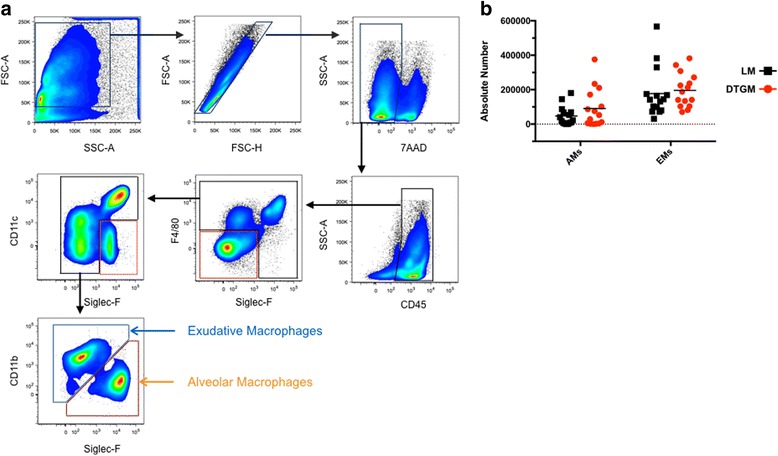
Flow cytometric discrimination of alveolar and exudative macrophages by surface marker expression. Representative FACS plots from an IAV-infected LM mouse at 10 dpi, which detail our 12-color flow cytometry gating strategy of single cell suspensions from BAL and enzyme-digested lung (**a**). Alveolar macrophages (AM) were designated as F4/80^+^ SiglecF^+^ CD11b^neg/dim^, whereas exudative macrophages (EM) were designated as F4/80^+^ SiglecF^neg/dim^ CD11b+. Supra-physiologic GM-CSF levels during IAV infection had no effect on the absolute number of either airway (BAL-recovered) AM or EM cell numbers at 10 dpi (**b**)

Alveolar macrophages have been shown to be necessary for protection from IAV [21–27]. GM-CSF is known to mediate the proliferation and differentiation of monocytes and macrophages; studies using constitutive expression or GM-CSF administration before IAV infection models both documented an increase in AM numbers [5, 6, 28]. Therefore we hypothesized that GM-CSF would protect SiglecF^+^ AMs from viral-induced depletion and would increase numbers of total airway (BAL-recovered) macrophages. To investigate this immune cells were characterized and enumerated in single cell suspensions of BAL and lung enzymatic digests by multi-parameter flow cytometry using a 12-color panel of macrophage and granulocyte surface markers (Fig. 4a). We specifically focused on the two predominant airway macrophage populations present during active IAV infection: F4/80^+^, CD11b^neg/dim^, SiglecF^+^ cells to discriminate alveolar macrophages (AMs) and F4/80^+^, CD11b^+^, SiglecF^neg/dim^ cells that have been termed exudative macrophages (EMs) [29]. Our typical yield of AMs recovered from BAL fluid of an uninfected mouse is approximately 600,000 cells. At 10dpi, at the height of the inflammatory response to IAV, the number of AMs recovered was much lower, and GM-CSF overexpression did not serve to increase this number (Fig. 4b). In contrast, EMs become the predominant airway macrophage during IAV infection at this time point, but again, GM-CSF overexpression did not affect EM cell numbers (Fig. 4b).

### Transcriptome analysis of airway macrophages during IAV infection

**Fig. 5:**
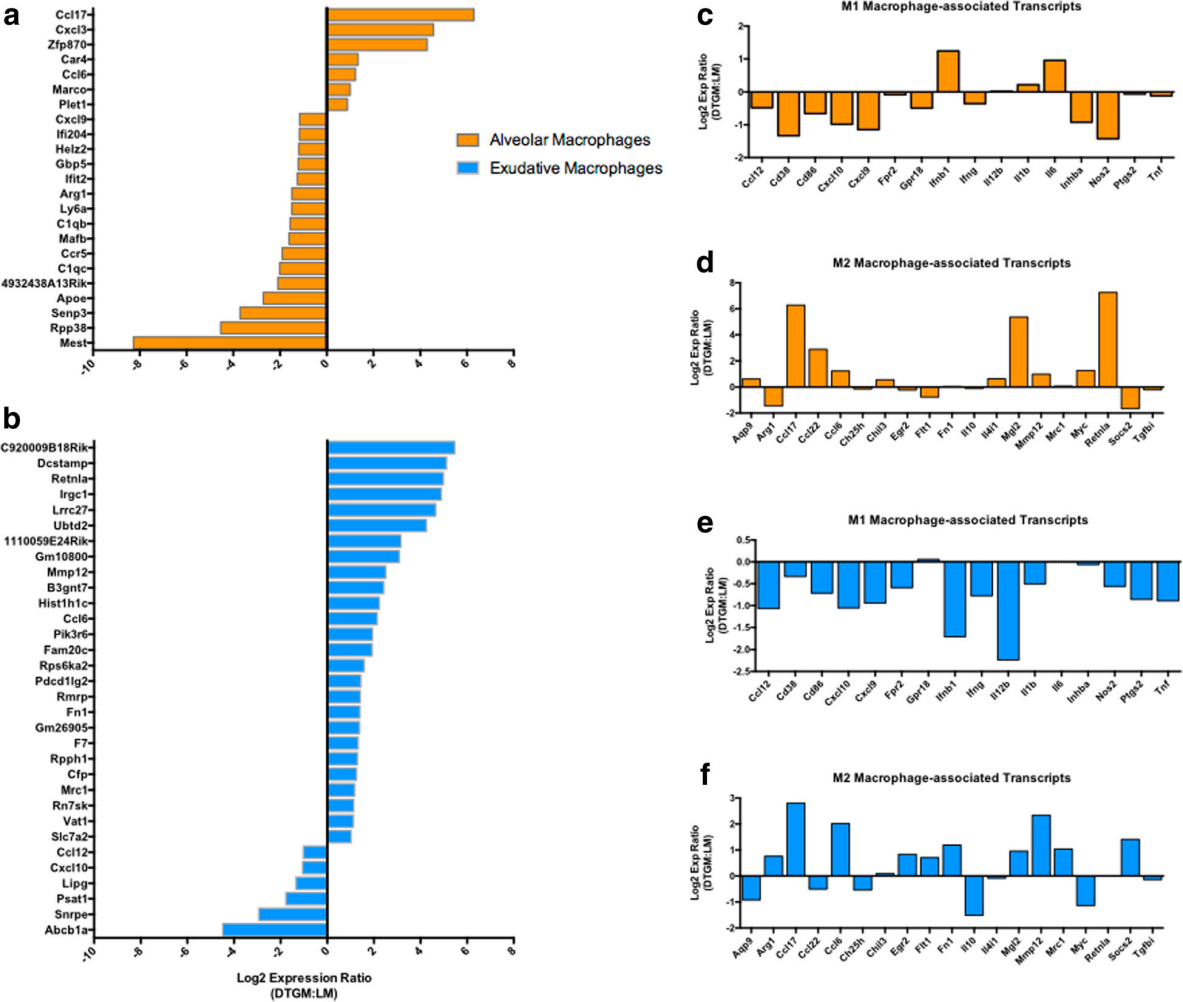
Characterization of the changes in transcriptome patterns of airway macrophages during IAV infection. BAL airway macrophages were sorted using the gating strategy described in Fig. 4a and next generation RNA-sequencing was used to profile the complete transcriptome data of AMs (**a**, orange bars) and EMs (**b**, blue bars) at 8 dpi, the time point at which the survival curves diverge (n = 5 mice per group). The effect of supraphysiologic GM-CSF levels on each of the 43,628 sequenced macrophage genes was examined: differential gene expression was determined using with transcripts having a q-value <0.2 being included. The relative expression of each transcript was calculated using the equation, Log2 Expression Ratio (DTGM:LM) = Log_2_ $\Big(\overline{X}$ transcript^DTGM^) - Log_2_ ($\overline{X}$ transcript^LM^), and the differential expression of transcripts is shown. To investigate the impact of GM-CSF on M1/M2 macrophage polarization, the Log2 Expression Ratios were plotted against known M1 and M2 macrophage-associated transcripts from AMs (**c**, **d**) and EMs (**e**, **f**)

While GM-CSF overexpression did not change the number of macrophages, we hypothesized that it changed their phenotype. This is not a new concept as the primary function of GM-CSF on AMs is to induce differentiation and activation [4, 30, 31]. Despite attempting to discriminate the macrophage populations by multiple cell surface markers, we could not distinguish the IAV-responding macrophages further than alveolar and exudative macrophages as described in Fig. 4a. Therefore, we sought to determine whether GM-CSF affected the transcriptomes of the AM and EM populations independently by first FACS-sorting the airway macrophages. FACS-sorted airway macrophages from BAL fluid were obtained at 8 dpi, the time point where the survival curves of the DTGM and LM mice begin to diverge, and next generation RNA-sequencing was performed on the sorted AM and EM populations. Using an unbiased approach, we identified the transcripts that were significantly affected by GM-CSF over-expression during IAV infection by comparing the mean value of each transcript from the DTGM and LM groups. For direct comparisons, transcripts that had a mean value of zero (0) FKPM in one of the groups were not analyzed. Of the 43,628 genes available in the reference genome, in the AM population 23 transcripts were significantly different between the groups with GM-CSF over-expression leading to up-regulation of the chemokines *Ccl17*, *Cxcl3*, and *CCL6*, and the down-regulation of *Cxcl9*, and *Arg1*, the prototypic marker of M2 macrophage polarization (Fig. 5a) [32]. In comparison to AMs, in EMs GM-CSF induced more transcripts than it inhibited. Only six transcripts were down regulated by GM-CSF including *Lipg*, *Cxcl10* and *Ccl12*, while GM-CSF overexpression induced multiple transcripts in EMs including *Dcstamp*, *Retnla*, *Irgc1*, *Mmp12*, and *Ccl6* (Fig. 5b). Our unbiased analysis demonstrated that GM-CSF overexpression during IAV led to the up-regulation of some transcripts associated with M2 macrophages including matrix metalloprotease 12, MMP12, and CCL17, and the down-regulation of some M1 macrophage-associated transcripts such as CXCL9 and CXCL10. Therefore we examined the effect of GM-CSF on multiple canonical and novel macrophage polarization markers [33]. Interestingly, while GM-CSF tended to down-regulate M1 transcripts and up-regulate M2 transcripts, this effect was not absolute in either AMs or EMs (Fig. 5c-f).

### BAL protein validation of RNAseq data

**Fig. 6:**
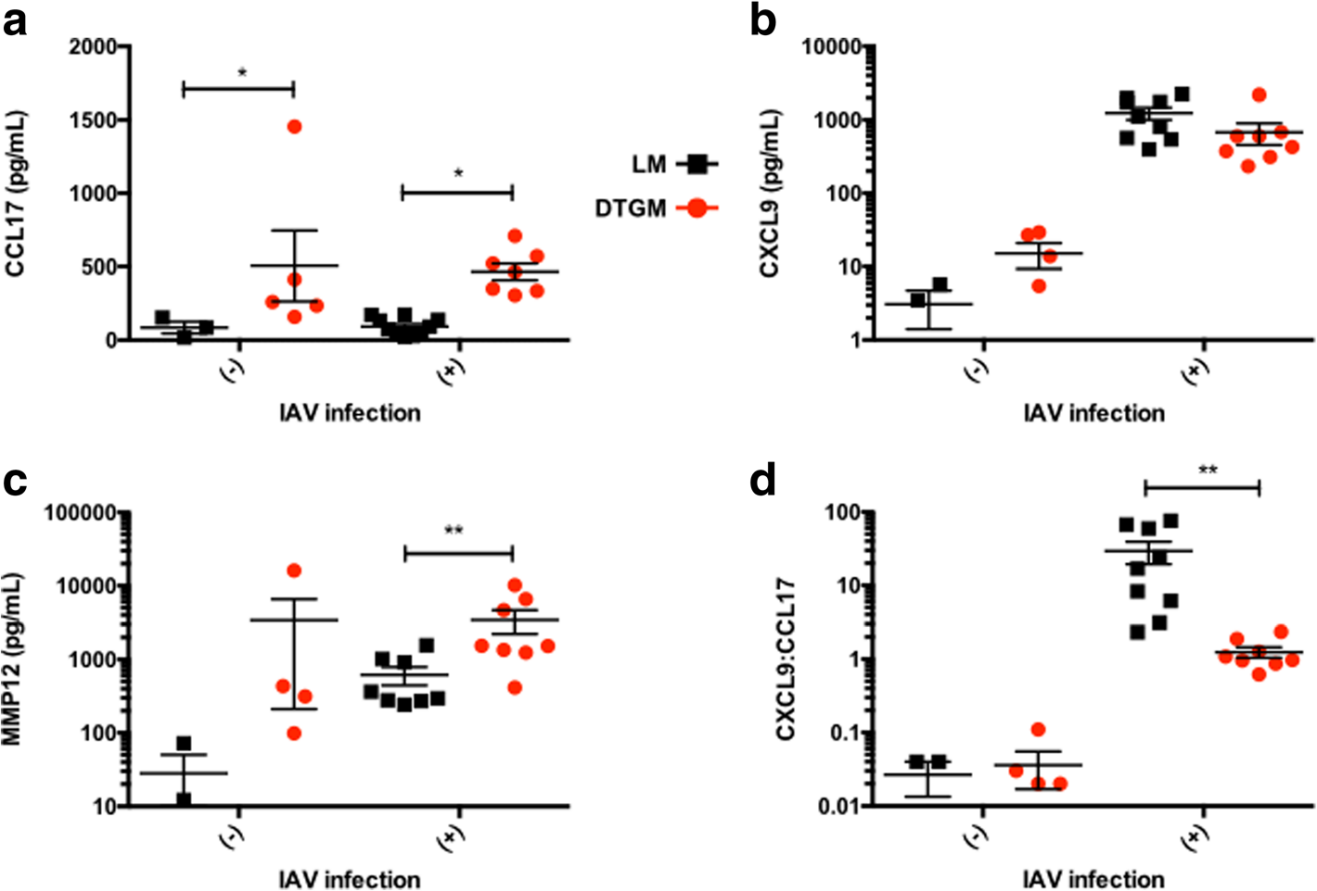
Effect of GM-CSF overexpression on airway levels of CCL17, CXCL9 and MMP12. Mouse CCL17 (**a**), CXCL9^#^ (**b**), and MMP12^#^ (**c**) were measured by ELISA in BAL fluid from doxycycline-treated LM (black) and DTGM (red) uninfected and IAV-infected (10 dpi) mice. Furthermore, the ratio of CXCL9:CCL17^#^ in each BAL sample was determined to examine the relative effect of supraphysiologic GM-CSF levels on macrophage chemokine polarization (**d**). Results from three independent experiments. (^#^Please note the log_10_ scale, *p < 0.05, **p < 0.005)

To validate these macrophage transcript differences we measured the chemokines CCL17 and CXCL9, and the M2-associated metalloprotease, MMP12, in BAL fluid by ELISA. CCL17 was significantly induced by GM-CSF (not only during IAV infection, but also when GM-CSF was induced in the absence of IAV (Fig. 6A). In comparison, negligible amounts of CXCL9 were present in uninfected mice regardless of GM-CSF induction, whereas with IAV infection GM-CSF overexpression there was a trend toward decreased expression (Fig. 6B, *p* = 0.09). The concentration of MMP12 was approximately 6-fold higher in GM-CSF overexpressing mice at 10 dpi as compared to LMs (Fig. 6C, *p* < 0.01). We also examined the ratio of the two chemokines (CXCL9: CCL17) as an intrinsic property of the BALF to probe macrophage polarization by chemokine expression and supra-physiologic GM-CSF levels significantly decreased this ratio more than ten-fold in IAV-infected mice (Fig. 6D, *p* < 0.005).

### Ingenuity pathway analysis of GM-CSF altered transcripts in airway macrophages

Lastly, we attempted to determine which signaling pathways were affected by GM-CSF overexpression during IAV infection by analyzing our transcriptomes with Ingenuity Pathway Analysis (IPA) software (Qiagen). We analyzed the effect of GM-CSF overexpression on the mean log_2_ expression ratios for the 5% most expressed genes in each of the macrophage type groups (AM vs. EM). The IPA software allows the construction of an upstream analysis that calculates the likelihood that an upstream regulator is involved given the gene set provided (*p*-value of overlap), as well as a composite score of activation depending on the state of downstream genes being increased or decreased in quantity (activation z-score). For both the upstream regulator analysis, and the subsequent canonical pathway analysis, we used a stringent p-value of overlap cutoff of 1E-10.

IPA predicted that GM-CSF activates (Table 1A) several upstream regulators of signaling pathways in both AMs and EMs including IL-10 receptor alpha (IL10RA), transcription factor tripartite motif-containing 24 (TRIM24), and the atypical chemokine receptor 2 (ACKR2). Conversely, IPA predicted that GM-CSF over-expression inhibited multiple inflammatory signaling pathways in both AMs and EMs including interferon regulatory factor 3 (IRF3), IRF7, interferon gamma (IFNG), interferon alpha/beta receptor (IFNAR), TIR domain-containing adapter molecule 1 (TICAM1, or TRIF), signal transducer and activator of transcription 1 (STAT1), rapamycin-insensitive companion of mammalian target of rapamycin (RICTOR), toll-like receptor 4 (TLR4), DExD/H-box helicase 58 (DDX58, or retinoic acid-inducible gene 1 [RIG-1]), and inhibitor of nuclear factor kappa-B kinase subunit beta (IKBKB).

**Table 1:**
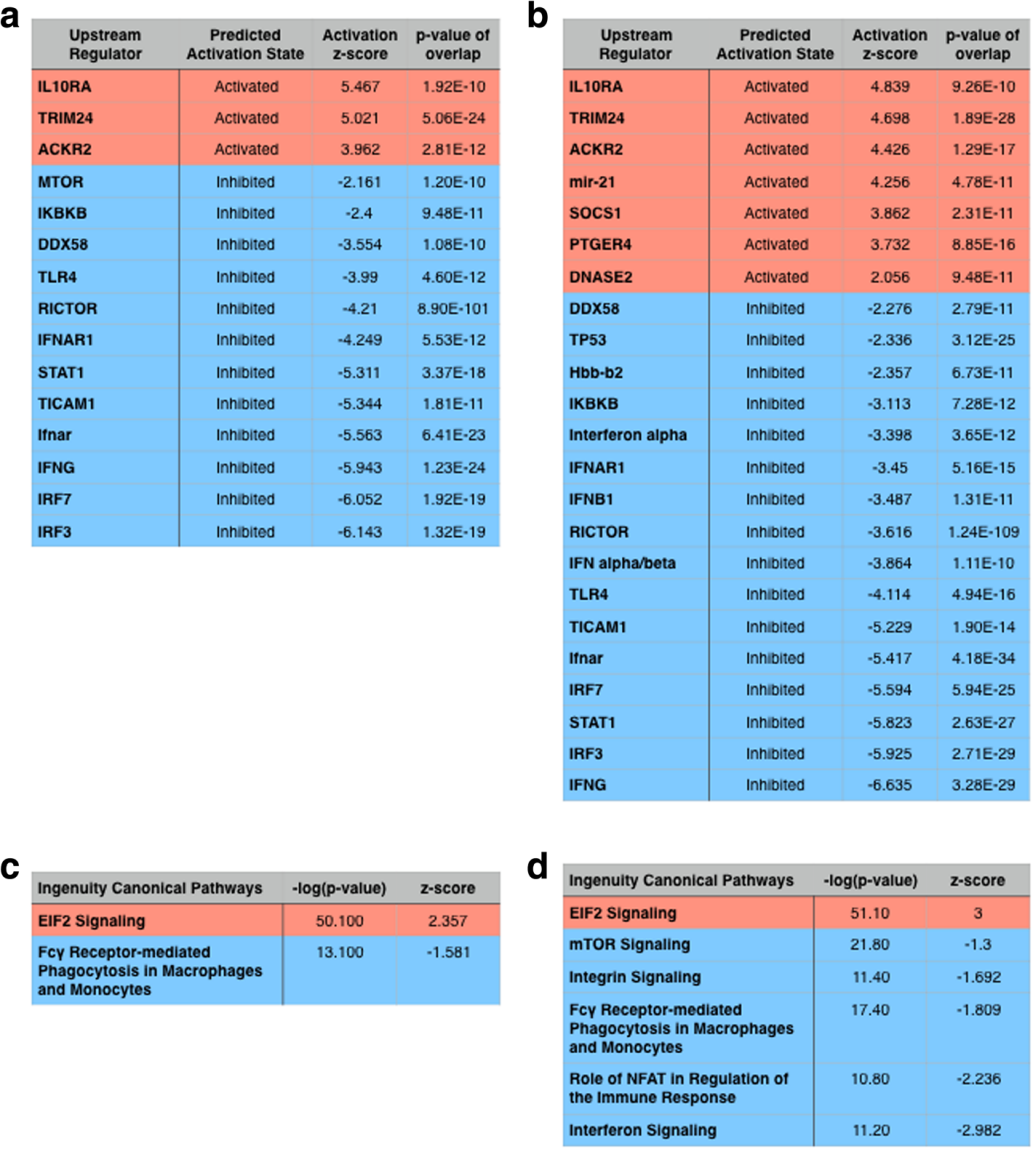
Ingenuity Pathway Analysis predictions of the effects of supra-physiologic levels of GM-CSF on airway macrophages during IAV. BAL airway macrophages were sorted and RNA-sequencing was performed to compare the gene expression between IAV-infected LM (*n* = 5 mice) and DTGM (*n* = 5 mice) treated with doxycycline at 8 dpi. Using the means of each group, the 5% (2181 genes) most expressed transcripts from each of the genotypes, DTGM and LM, were analyzed using Qiagen’s Ingenuity Pathway Analysis (IPA) software. IPA was used to identify differential upstream regulators between AMs (A) and EMs (B) of DTGM and LM mice, and upstream regulators were included in the table if their *p*-value of overlap was <1E-10 and the activation z-score was < −2 or > +2. IPA was also used to identify differential effects of GM-CSF on canonical pathways of AMs (C) and EMs (D). Ingenuity canonical pathways were included in the table if their -log(*p*-value) was >10 and the z-score of pathway activation was < −2 or > +2

In terms of canonical pathway analysis one pathway, "eukaryotic initiation factor 2 (eIF2) signaling", was activated in both AMs and EMs, whereas "Fc-γ receptor-mediated phagocytosis in macrophages and monocytes" was inhibited in both populations (Table 1C, D). “Interferon signaling” was inhibited in EMs (Table 1D), and trended towards significance in the AM population [−log(p-value) 7.66, z-score − 1.5], even though the levels of type I, II and III interferons were unchanged in BAL fluid from DTGM as compared to WT mice (Additional file 6: Figures S4 A-C).

## Discussion

In this study we examined the effect of elevated GM-CSF levels during IAV infection on clinical, lung physiologic and biochemical markers in a mouse model, and then used RNA-sequencing to ascertain the differential effects of elevated GM-CSF levels on the transcriptomes of the two predominant airway macrophages present during the peak of IAV infection. Our finding that elevation of airway GM-CSF during active IAV infection confers protection from mortality from IAV is novel. Multiple preclinical mouse studies have described the observations that the absence of GM-CSF increases susceptibility to IAV [3, 4, 25], while supra-physiologic levels of GM-CSF achieved by constitutive overexpression or exogenous administration are beneficial [5, 6, 28]. Importantly, however, the publications that have demonstrated positive effects of supra-physiologic levels of GM-CSF against IAV infection have used either constitutive expression models [3–5] or have administered GM-CSF either before [6, 28] or on the day of infection [5]. To our knowledge this is the first description of the use of a therapeutic model of GM-CSF wherein it is “administered” to the airways well after establishment of the infection (+3 dpi) and still confers protection.

GM-CSF over-expression led to an increase in macrophage expression and BAL fluid levels of CCL17 and MMP12, whereas a decrease in CXCL9 or monokine induced by gamma interferon (MIG). These protein data, in addition to our macrophage transcriptome data, suggest that high levels of GM-CSF push the typically classically activated M1-like monocytes/macrophages in the lung during IAV towards an M2-like phenotype. Interestingly, a recent investigation showed that the presence of M1-like monocytes are a major determinant of IAV pathogenicity in patients and strengthened this notion with a mouse model demonstrating that adoptive transfer of M2 as opposed to M1 macrophages results in better outcomes [34]. The observation that GM-CSF is pushing macrophages towards an M2-phenotype is in stark contrast to a large body of in vitro literature that defines M1 monocytes/macrophages as being induced by GM-CSF, whereas M2 monocytes/macrophages are differentiated by macrophage colony-stimulating factor (M-CSF) [35–37]. On the other hand, alveolar macrophages from GM-CSF-deficient csf2−/− mice exhibit a mixed M1/M2 phenotype, not a strictly M2 phenotype as in vitro data would suggest [38]. And our data also suggests that the polarization was not at all absolute: e.g., in AMs, GM-CSF led to lower transcript levels of the prototypic M2 macrophage marker, Arg1 (Fig. 5a). Thus, while the M1/M2 macrophage polarization schema has been helpful [39, 40], perhaps a more nuanced view of macrophage polarization [41], where their intrinsic differentiation plasticity allows them to attend to specific needs of their local immune environment [42], could explain these results. IPA also predicted the activation of the IL-10 receptor alpha-chain in both AMs and EMs. Given that IL-10 levels in BAL fluid were not elevated in DTGM as compared WT mice (Additional file 6: Figure S4D), it is possible that GM-CSF over-expressing during IAV somehow potentiates IL-10 signaling in the lung microenvironment.

The role of interferons during IAV infection is also nuanced. While it has been shown using IFNAR^−/−^ and IFNGR1^−/−^ mice that interferon signaling is necessary for protection from IAV [43], it is possible that this requirement only extends to epithelial cells. Interferon-γ may not be necessary during IAV infection and may in fact be detrimental, e.g., nitrogen oxide synthase 2 deficient (NOS2−/−) mice are more protected from IAV [44], and Sun and Metzger demonstrated that treatment with an anti-IFNγ mAb clone XMG1.2 had little effect on the course of the viral infection, but inactivation of IFN-γ protected against secondary bacterial pneumonia [45]. Recently, Califano et al. showed that IFNγ^−/−^ mice on both the Balb/c and C57BL/6 backgrounds demonstrated improved survival to lethal IAV infection [34]. In their model, IFNγ serves to restrict protective innate lymphoid cell group 2 (ILC2) function, whose production of IL-5 and amphiregulin may improve lung barrier function. Another group has also demonstrated that GM-CSF can induce amphiregulin in a smoke model of COPD followed by IAV infection [46], however our data (Fig. 3c) suggest that pretreatment with GM-CSF is necessary for this effect on amphiregulin levels. Furthermore, our GM-CSF over-expression is started after IAV infection, amphiregulin levels at 10 dpi were not different in GM-CSF over-expressing mice, and therefore amphiregulin is likely not an active player in our model. It is possible that our inducible GM-CSF model may be replenishing GM-CSF that otherwise would be produced by ILC2s whose functions have been restricted by IFNγ [34].

Our data suggest that high levels of GM-CSF inhibit interferon signaling in airway macrophages, though the mechanism is not clear. Canonically, GM-CSF signaling acts through JAK2/STAT5 [47], though the beta-chain itself can activate NF-kB, and this activation is dependent on TNFR-associated factor 6 (TRAF6) [48, 49], an E3 ubiquitin ligase with multiple immune functions [50]. Interestingly, our upstream analysis predicts that GM-CSF activates TRIM24, (aka TIF1α), a negative regulator of interferon signaling that acts by binding the retinoic acid-responsive element of the *Stat1* promoter [51], thus inactivating multiple interferon pathways. TRIM24 is also an E3-ubiquitin ligase, and the tumor suppressor protein, p53, serves as a ligand for both ligases: TRIM24 targets p53 for degradation [52] while TRAF6 restricts p53 mitochondrial translocation [53]. Furthermore, a recent microarray study examining the relative pathogenicity of a mouse adapted strain of IAV (MA-CA/04) described negative inhibition of TRIM24 and early sustained interferon responses as important factors [54]. However, we detected only very low levels of *Trim24* transcripts in our sorted airway macrophages, but GM-CSF over-expression did lead to increased expression of another TRIM family member, Trim16, that also acts as a E3 ubiquitin ligase that can heterodimerize with other TRIMs [55]. Future studies are needed to determine the exact cellular signaling pathways linking GM-CSF and interferon.

GM-CSF enhanced exudative macrophage expression, and 8 dpi BAL fluid levels of MMP12, or macrophage elastase, which is best known for its requirement for the development of smoke-induced emphysema in mice [56]. However, it may also regulate acute inflammatory responses by proteolysis of chemokines [57], and through its divergent effects on IFN-α signaling depending on its intracellular (activating) vs. extracellular (inactivating) localization [58]. A recent report demonstrated in two separate mouse models inflammation (peritonitis and arthritis) that macrophages resolve inflammation through multiple mechanisms via MMP12 including dampening neutrophil infiltration, clearing actin and fibrin from NETs, terminating complement activation, and by activating prothrombin thus exhibiting procoagulant activity [59]. CD4+ T cells and STAT4/6, at least in a mouse model of pneumocystis pneumonia, are necessary for M2 macrophage MMP12 expression and RELM-α and CCL17 production [60]. While our data suggest that GM-CSF may block M1-like polarization in the lung during IAV infection, it is not yet clear what in the lung microenvironment could promote M2-like macrophage responses. Recently it was shown that macrophage polarization may be pushed towards a IL-4 dependent pathway in the lung and liver by the presence of surfactant protein A (SP-A) and complement component C1q, respectively [61]. The relationship between supraphysiologic GM-CSF levels and SP-A during IAV infection remains to be investigated and will be the subject of future studies.

Our current model of GM-CSF induction on the wild-type background differs from our previous work using the inducible model generated on the GM-CSF knockout (*Csf2*^*−/−*^) genetic background [4]. The present study is not confounded by prior immaturity of AMs and defective surfactant catabolism, nor potential defects in migratory dendritic cell subsets, NK cells, and other myeloid cells outside the alveolar compartment in the lung and in other tissues of *Csf2*^−/−^ mice [62–65], or disruption of GM-CSF secretion by immune and non-immune cells that may elaborate GM-CSF in response to infection. Studies in *Csf2*^−/−^ /SPC-GM mice, in which T2AECs express high levels of GM-CSF constitutively, came to disparate conclusions as to the role of AMs, dendritic cells and epithelial cells [3, 5] in host resistance to IAV infection. However, the life-long overexpression of GM-CSF in the SPC-GM^+/+^ model results in non-physiological proliferation of both T2AEC cells and AMs [66] that obscures assessment of temporal responses to IAV. The SPC-GM^+/+^ model also illustrates that prolonged lung exposure to supraphysiologic levels of GM-CSF leads to desquamative interstitial pneumonia (DIP) [4]. We did not observe any similar findings of DIP in our model, but this is not surprising given our model creates only a temporary doxycycline-induced overxpression, and the overriding inflammatory effects of IAV infection likely masks any differences. In our model, the ability of supra-physiologic levels of GM-CSF to beneficially alter disease progression after IAV infection delineates a time frame for possible future therapeutic intervention to arrest development of acute lung injury. In this regard, administration of GM-CSF in humans has shown promise in the treatment of ARDS [67]. Concentration-dependent signaling via the GM-CSF receptor affecting differentiation, proliferation, activation, and function of different effector cells has been studied extensively [68–71].

## Conclusions

Our data demonstrate that in vivo high airway levels of GM-CSF profoundly rescue mice from lethal influenza pneumonia. While in vitro GM-CSF is canonically described as an M1-polarizing cytokine, our data demonstrates that in vivo, during IAV infection, GM-CSF instead temporizes the type II interferon-induced M1 polarization of airway macrophages. The exact mechanism through which high levels of GM-CSF block M1-macrophage polarization is still not known, and is the focus of our ongoing research.

## Additional files

Additional file 1: Table S1. All protein concentration measurements were made as described in the manuscript text using the reagents and kits listed. (TIFF 3075 kb)

Additional file 2: Table S2. Multi-parameter flow cytometry was utilized to characterize the alveolar and exudative macrophages as shown in Fig. 4. All monoclonal antibodies were purchased from either AbD Serotec, BD Bioscience or eBioscience, respectively. (TIFF 3075 kb)

Additional file 3: Figures S1A-D. Lethal dose 50% (LD50) determination of influenza virus strain A/Puerto Rico/8/1934 (PR8) in female (A, B) and male (C, D) mice, demonstrating an LD50 of 728 vs. 3728 fluorescent focus units (FFU) in female and males, respectively. (ZIP 87 kb)

Additional file 4: Figure S2A and B. Characterization of the Double Transgenic GM-csf (DTGM) mouse model. In the absence of influenza A virus infection, GM-CSF levels (A) in bronchoalveolar lavage (BAL) fluid was low, near the limit of detection in littermate (LM) and DTGM mice. Upon influenza A virus infection DTGM mice without doxycycline-induction (DTGM noDox) demonstrate "leakiness" that corresponds to the peak of type II interferon levels at days 7-8 post-infection. DTGM +Dox mice demonstrate supra-physiologic levels of GM-CSF in BAL fluid at all time points after induction. DTGM mice were less susceptible to IAV infection (B) even in the absence of doxycycline induction, whereas doxycycline administration to LM mice had no effect. (ZIP 55 kb)

Additional file 5: Figure S3A and B. Measurement of serum proteins in BAL fluid. Elevated levels of GM-CSF neither affected the quantity of mouse albumin (A) nor IgM (B) in BAL fluid at 10 and 14 days post-infection. (ZIP 39 kb)

Additional file 6: Figures S4A-D. Characterization of the kinetics of BAL cytokines. Type I interferon (A), type II interferon (B), type III interferon (C), and IL-10 (D), were measured in BAL fluid from wild-type (WT, gray bars) or DTGM +Dox (red bars) mice by multiplex analysis (Luminex, https://www.luminexcorp.com) at the indicated time points. (ZIP 74 kb)
