# Supplementary figures and images for: GM-CSF overexpression after influenza a virus infection prevents mortality and moderates M1-like airway monocyte/macrophage polarization
Source: Respir Res. 2018 Jan 5;19:3. doi: 10.1186/s12931-017-0708-5 (PMC5756339; doi:10.1186/s12931-017-0708-5)

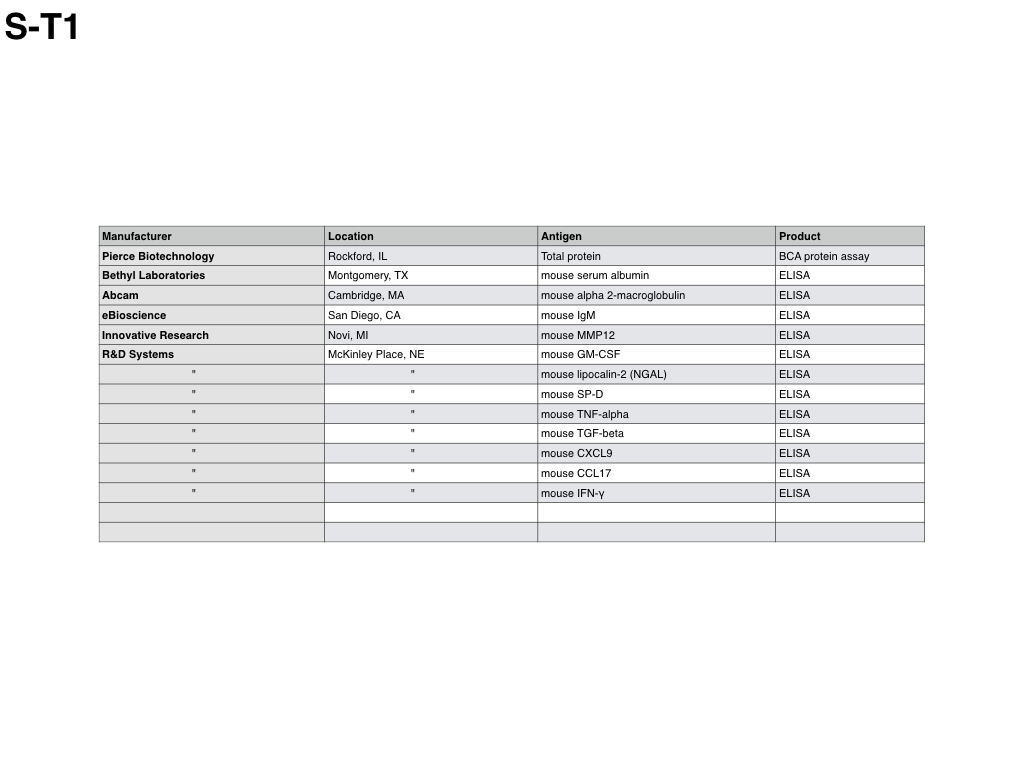

Supplement: Supplementary file 1 — All protein concentration measurements were made as described in the manuscript text using the reagents and kits listed. (TIFF 3075 kb) [file 12931_2017_708_MOESM1_ESM.tiff]

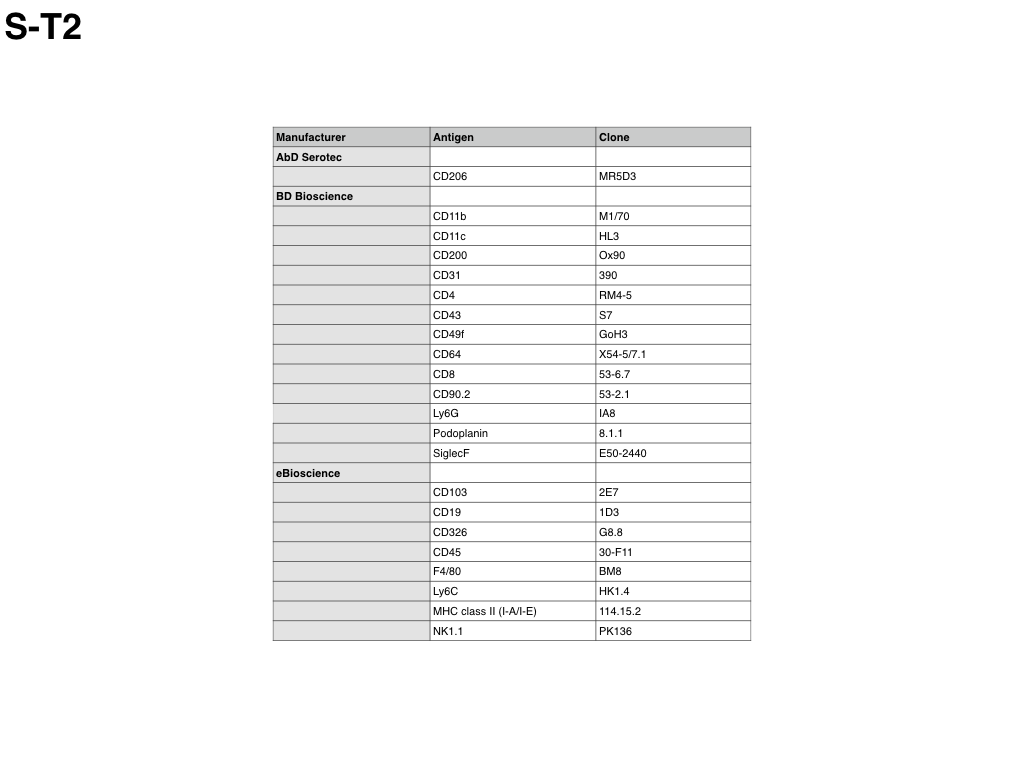

Supplement: Supplementary file 2 — Multi-parameter flow cytometry was utilized to characterize the alveolar and exudative macrophages as shown in Fig. 4. All monoclonal antibodies were purchased from either AbD Serotec, BD Bioscience or eBioscience, respectively. (TIFF 3075 kb) [file 12931_2017_708_MOESM2_ESM.tiff]

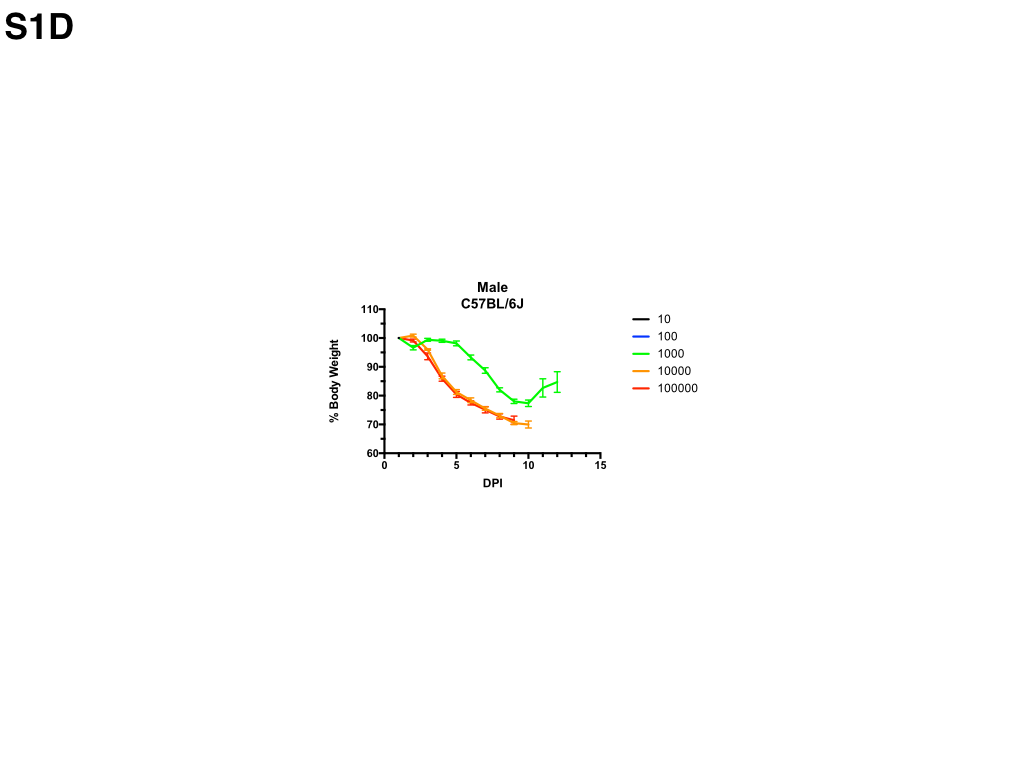

Supplement: Supplementary file 3 — Lethal dose 50% (LD50) determination of influenza virus strain A/Puerto Rico/8/1934 (PR8) in female (A, B) and male (C, D) mice, demonstrating an LD50 of 728 vs. 3728 fluorescent focus units (FFU) in female and males, respectively. (ZIP 87 kb) [file 12931_2017_708_MOESM3_ESM.zip › Figure Images.S1D.tiff]

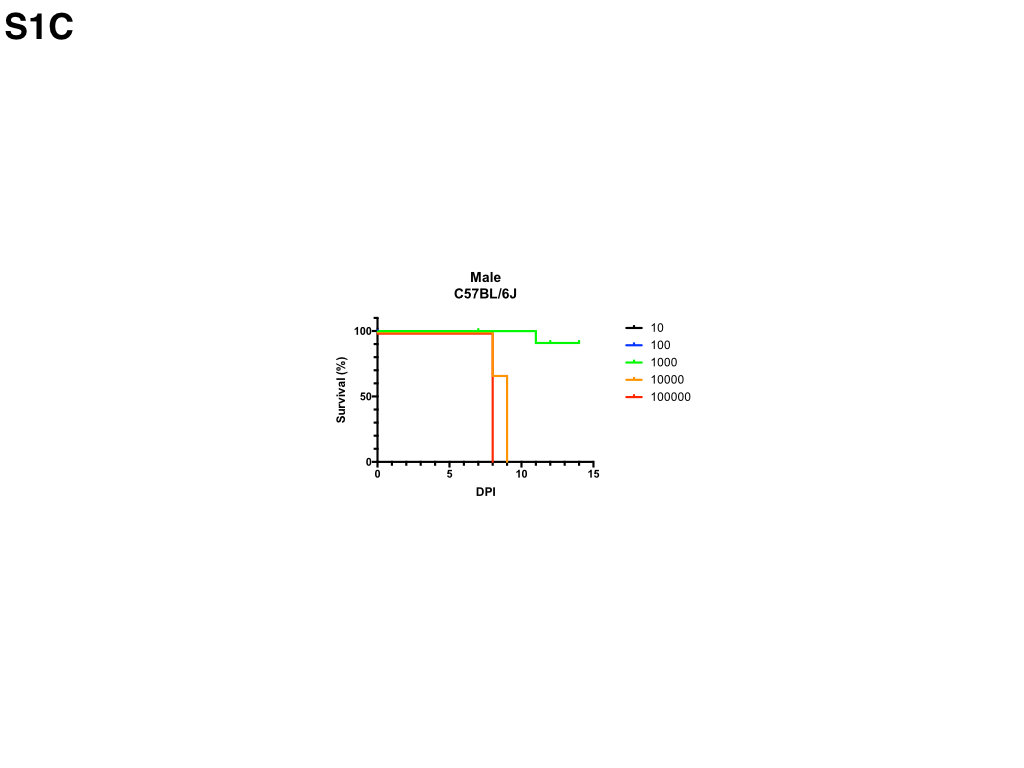

Supplement: Supplementary file 3 — Lethal dose 50% (LD50) determination of influenza virus strain A/Puerto Rico/8/1934 (PR8) in female (A, B) and male (C, D) mice, demonstrating an LD50 of 728 vs. 3728 fluorescent focus units (FFU) in female and males, respectively. (ZIP 87 kb) [file 12931_2017_708_MOESM3_ESM.zip › Figure Images.S1C.tiff]

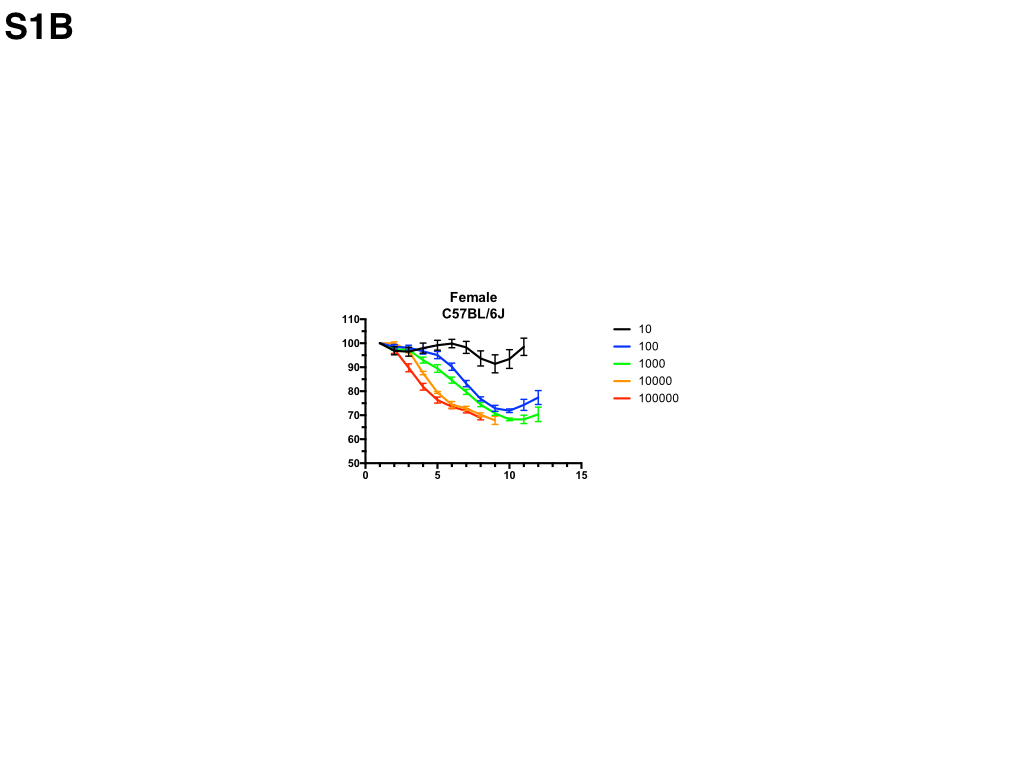

Supplement: Supplementary file 3 — Lethal dose 50% (LD50) determination of influenza virus strain A/Puerto Rico/8/1934 (PR8) in female (A, B) and male (C, D) mice, demonstrating an LD50 of 728 vs. 3728 fluorescent focus units (FFU) in female and males, respectively. (ZIP 87 kb) [file 12931_2017_708_MOESM3_ESM.zip › Figure Images.S1B.tiff]

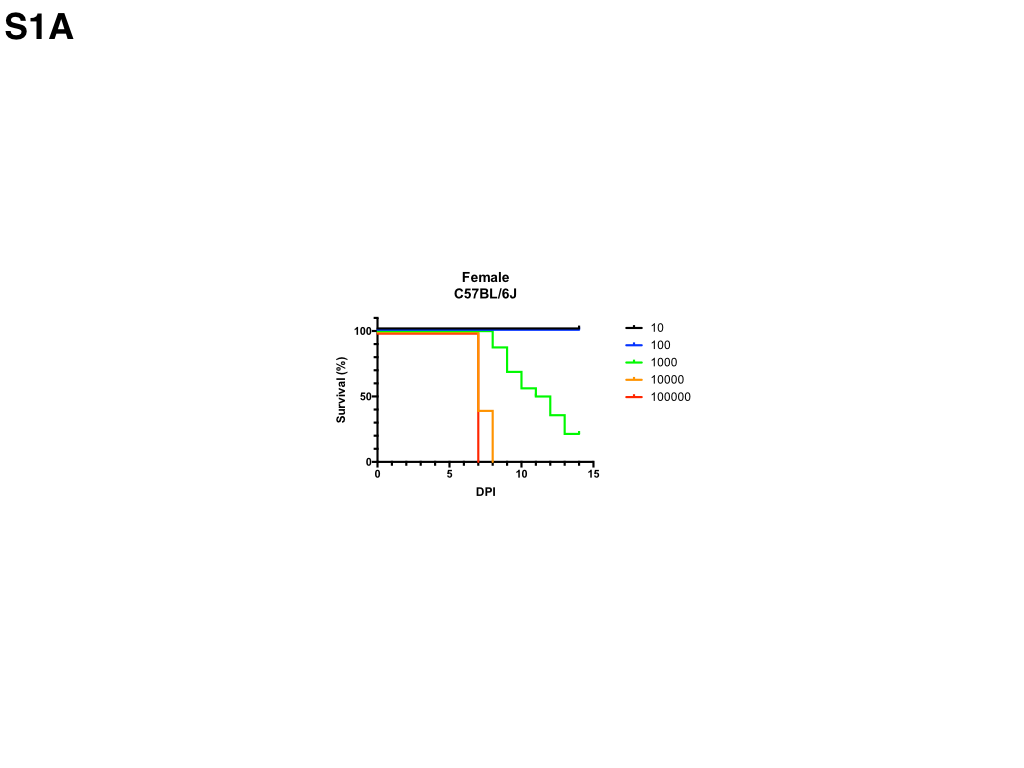

Supplement: Supplementary file 3 — Lethal dose 50% (LD50) determination of influenza virus strain A/Puerto Rico/8/1934 (PR8) in female (A, B) and male (C, D) mice, demonstrating an LD50 of 728 vs. 3728 fluorescent focus units (FFU) in female and males, respectively. (ZIP 87 kb) [file 12931_2017_708_MOESM3_ESM.zip › Figure Images.S1A.tiff]

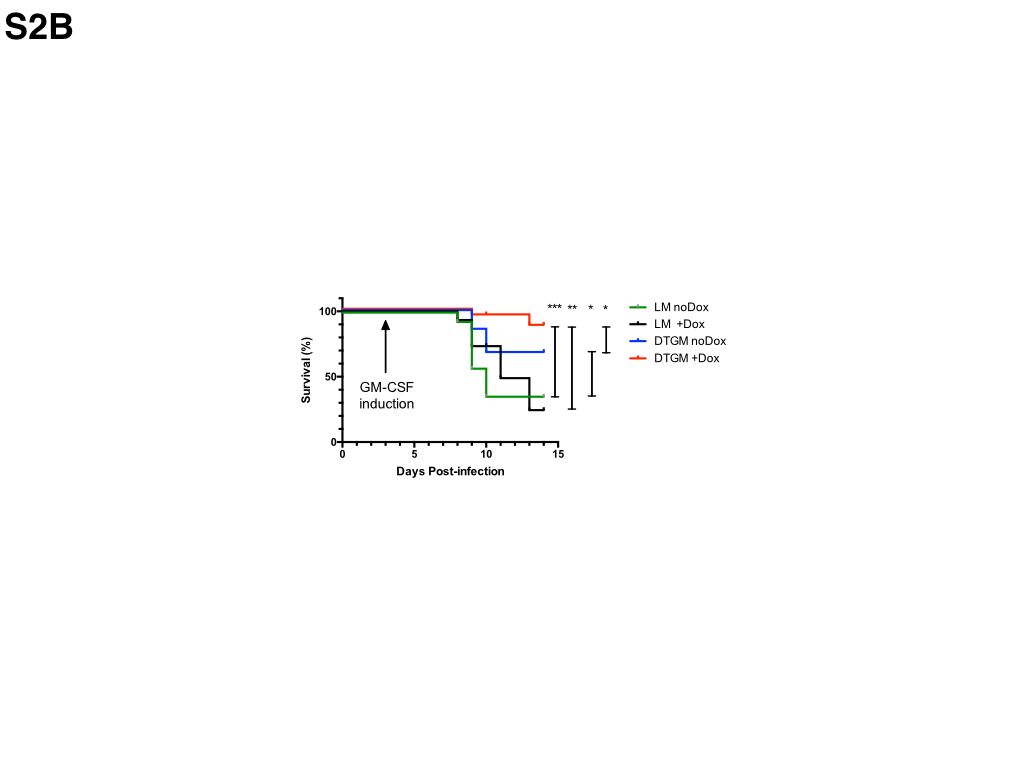

Supplement: Supplementary file 4 — Characterization of the Double Transgenic GM-csf (DTGM) mouse model. In the absence of influenza A virus infection, GM-CSF levels (A) in bronchoalveolar lavage (BAL) fluid was low, near the limit of detection in littermate (LM) and DTGM mice. Upon influenza A virus infection DTGM mice without doxycycline-induction (DTGM noDox) demonstrate "leakiness" that corresponds to the peak of type II interferon levels at days 7-8 post-infection. DTGM +Dox mice demonstrate supra-physiologic levels of GM-CSF in BAL fluid at all time points after induction. DTGM mice were less susceptible to IAV infection (B) even in the absence of doxycycline induction, whereas doxycycline administration to LM mice had no effect. (ZIP 55 kb) [file 12931_2017_708_MOESM4_ESM.zip › Figure Images.S2B.tiff]

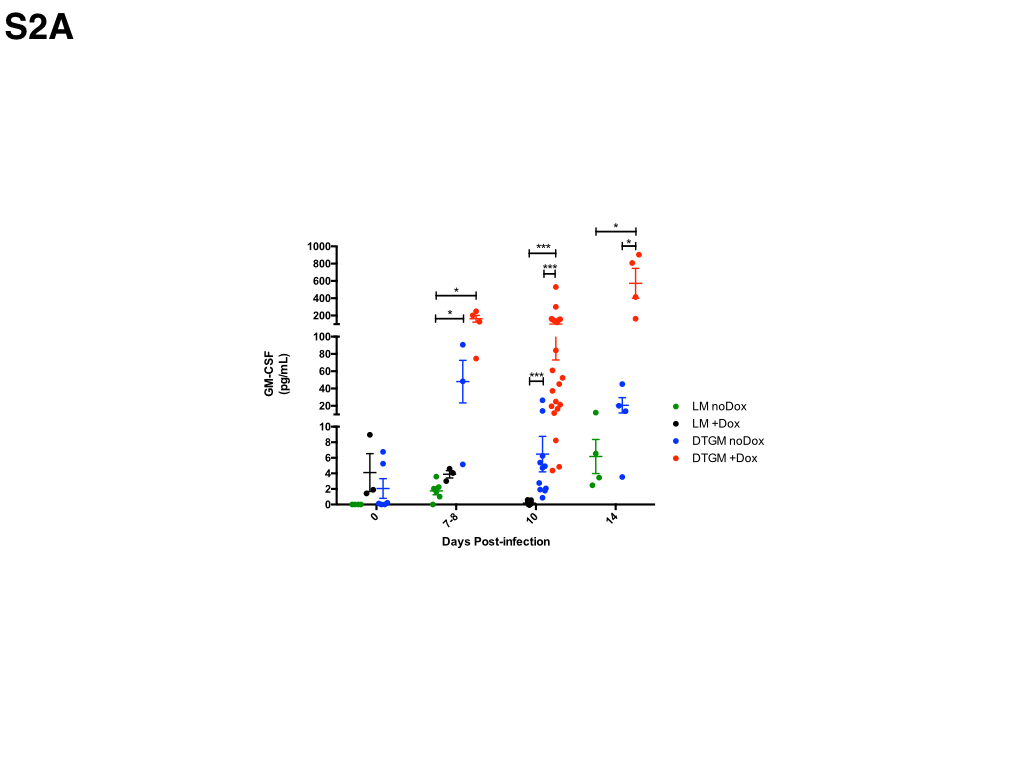

Supplement: Supplementary file 4 — Characterization of the Double Transgenic GM-csf (DTGM) mouse model. In the absence of influenza A virus infection, GM-CSF levels (A) in bronchoalveolar lavage (BAL) fluid was low, near the limit of detection in littermate (LM) and DTGM mice. Upon influenza A virus infection DTGM mice without doxycycline-induction (DTGM noDox) demonstrate "leakiness" that corresponds to the peak of type II interferon levels at days 7-8 post-infection. DTGM +Dox mice demonstrate supra-physiologic levels of GM-CSF in BAL fluid at all time points after induction. DTGM mice were less susceptible to IAV infection (B) even in the absence of doxycycline induction, whereas doxycycline administration to LM mice had no effect. (ZIP 55 kb) [file 12931_2017_708_MOESM4_ESM.zip › Figure Images.S2A.tiff]

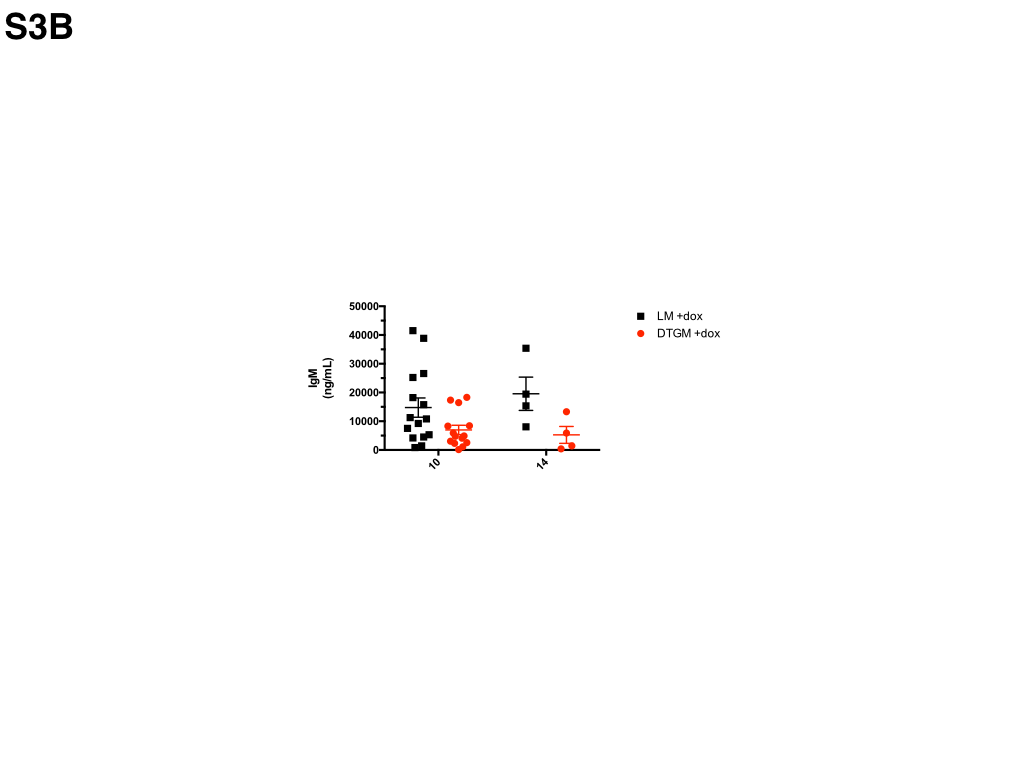

Supplement: Supplementary file 5 — Measurement of serum proteins in BAL fluid. Elevated levels of GM-CSF neither affected the quantity of mouse albumin (A) nor IgM (B) in BAL fluid at 10 and 14 days post-infection. (ZIP 39 kb) [file 12931_2017_708_MOESM5_ESM.zip › Figure Images.S3B.tiff]

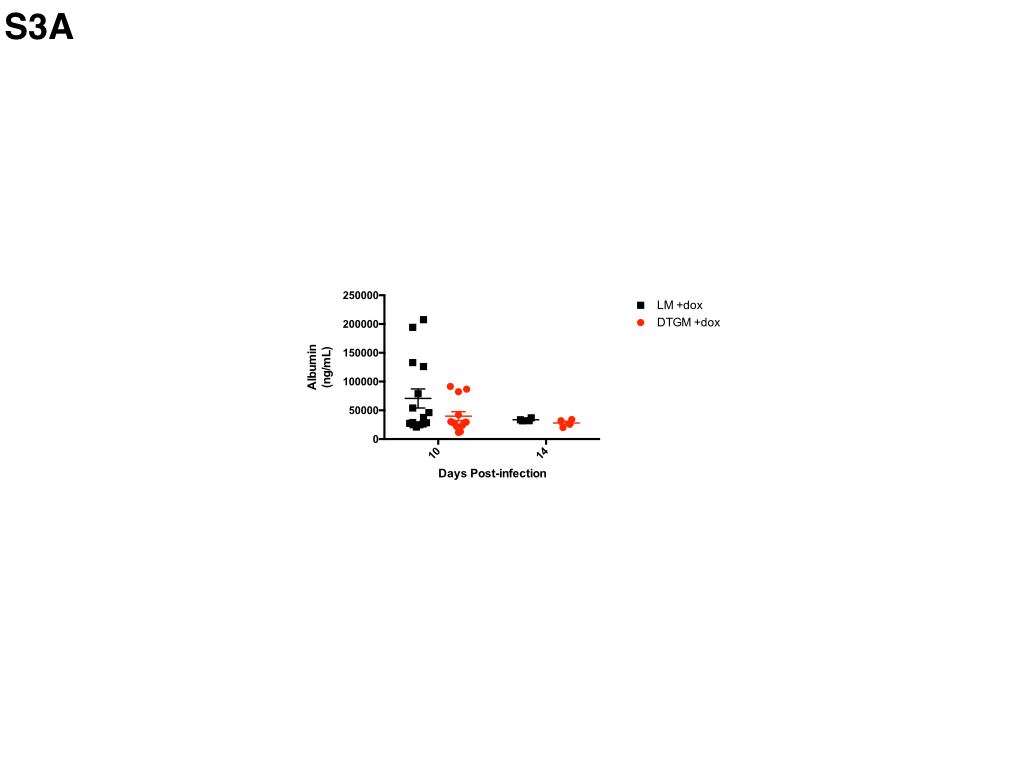

Supplement: Supplementary file 5 — Measurement of serum proteins in BAL fluid. Elevated levels of GM-CSF neither affected the quantity of mouse albumin (A) nor IgM (B) in BAL fluid at 10 and 14 days post-infection. (ZIP 39 kb) [file 12931_2017_708_MOESM5_ESM.zip › Figure Images.S3A.tiff]

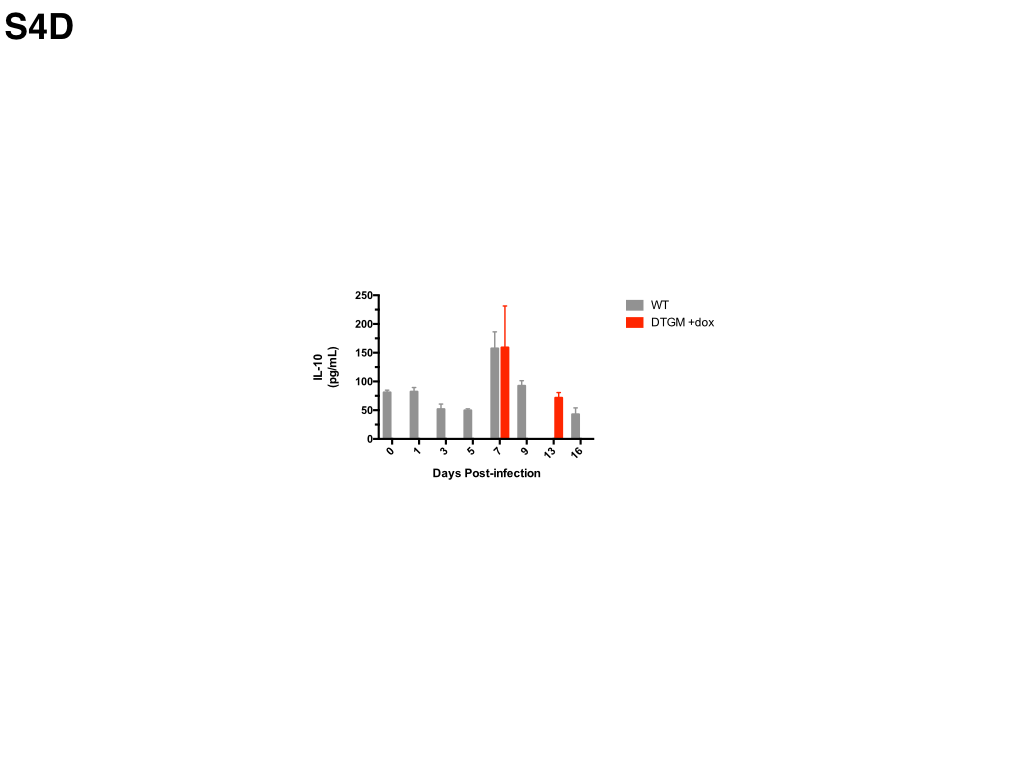

Supplement: Supplementary file 6 — Characterization of the kinetics of BAL cytokines. Type I interferon (A), type II interferon (B), type III interferon (C), and IL-10 (D), were measured in BAL fluid from wild-type (WT, gray bars) or DTGM +Dox (red bars) mice by multiplex analysis (Luminex, https://www.luminexcorp.com) at the indicated time points. (ZIP 74 kb) [file 12931_2017_708_MOESM6_ESM.zip › Figure Images.S4D.tiff]

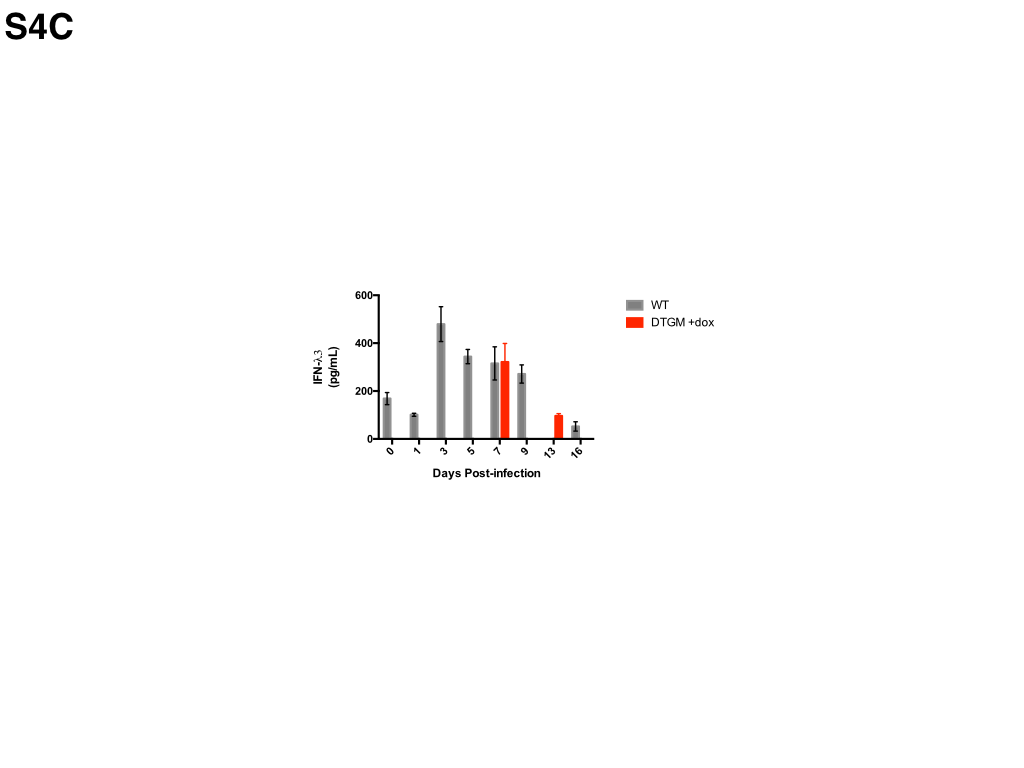

Supplement: Supplementary file 6 — Characterization of the kinetics of BAL cytokines. Type I interferon (A), type II interferon (B), type III interferon (C), and IL-10 (D), were measured in BAL fluid from wild-type (WT, gray bars) or DTGM +Dox (red bars) mice by multiplex analysis (Luminex, https://www.luminexcorp.com) at the indicated time points. (ZIP 74 kb) [file 12931_2017_708_MOESM6_ESM.zip › Figure Images.S4C.tiff]

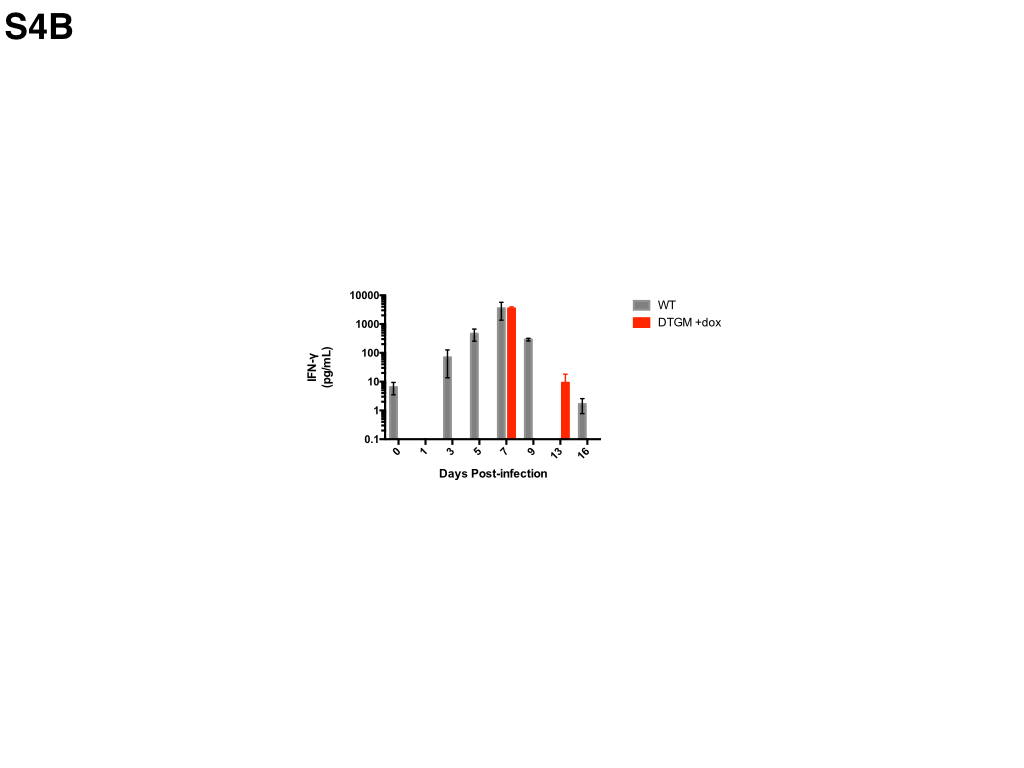

Supplement: Supplementary file 6 — Characterization of the kinetics of BAL cytokines. Type I interferon (A), type II interferon (B), type III interferon (C), and IL-10 (D), were measured in BAL fluid from wild-type (WT, gray bars) or DTGM +Dox (red bars) mice by multiplex analysis (Luminex, https://www.luminexcorp.com) at the indicated time points. (ZIP 74 kb) [file 12931_2017_708_MOESM6_ESM.zip › Figure Images.S4B.tiff]

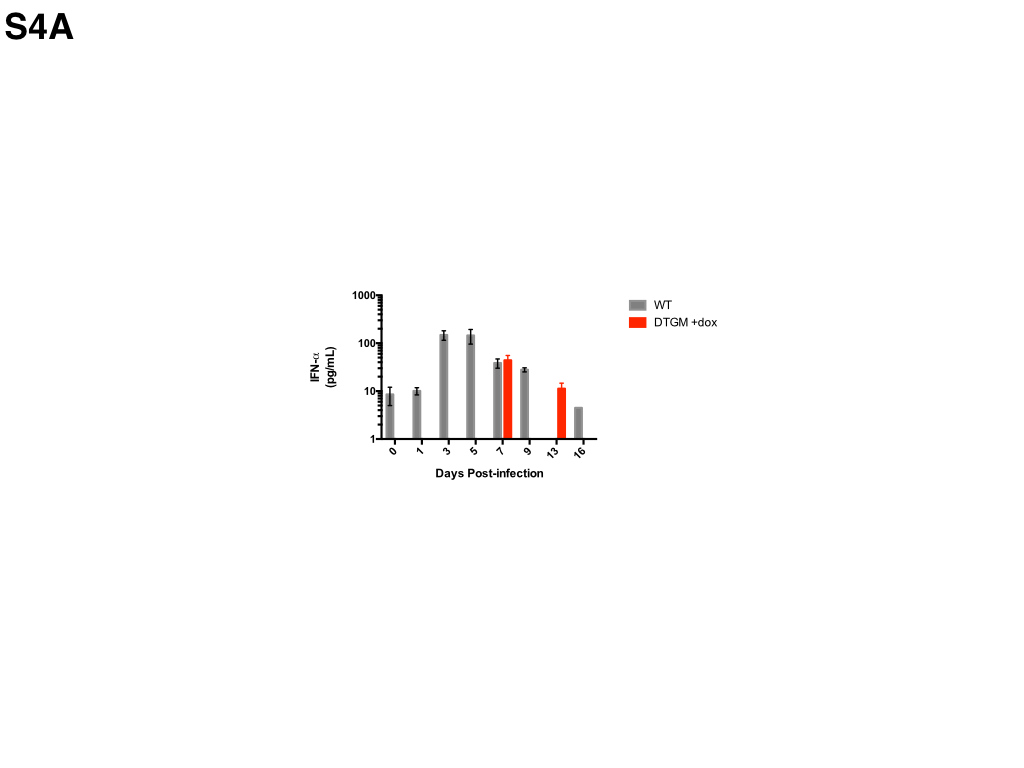

Supplement: Supplementary file 6 — Characterization of the kinetics of BAL cytokines. Type I interferon (A), type II interferon (B), type III interferon (C), and IL-10 (D), were measured in BAL fluid from wild-type (WT, gray bars) or DTGM +Dox (red bars) mice by multiplex analysis (Luminex, https://www.luminexcorp.com) at the indicated time points. (ZIP 74 kb) [file 12931_2017_708_MOESM6_ESM.zip › Figure Images.S4A.tiff]
